# Supplementary figures and images for: The Arabidopsis DNA Polymerase δ Has a Role in the Deposition of Transcriptionally Active Epigenetic Marks, Development and Flowering
Source: PLoS Genet. 2015 Feb 18;11(2):e1004975. doi: 10.1371/journal.pgen.1004975 (PMC4334202; doi:10.1371/journal.pgen.1004975)

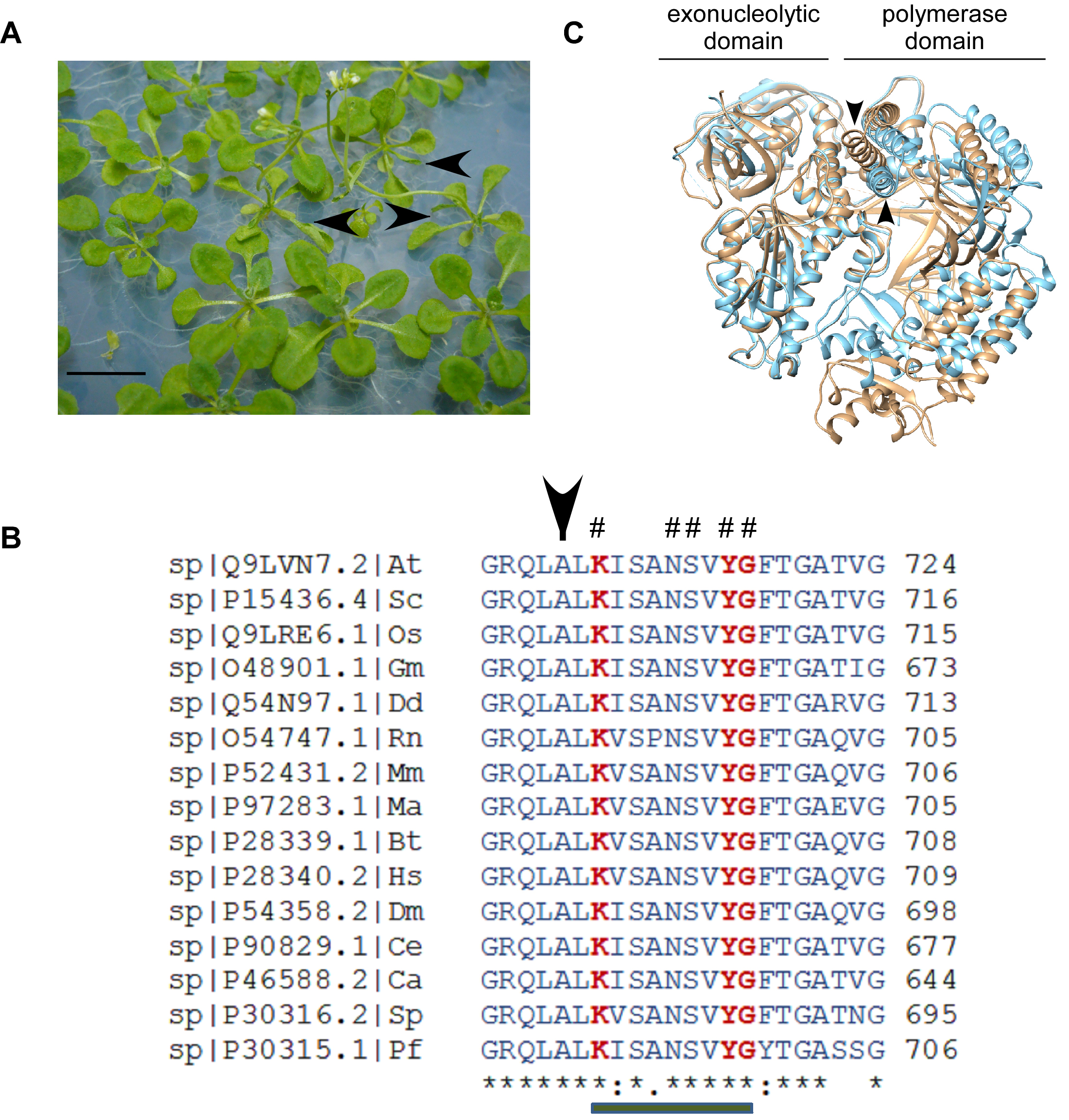

Supplement: S1 Fig — (A) The WT POLD1 sequence complements gis5 curly leaf phenotype. gis5 mutant plants were transformed with a plasmid containing the WT POLD1 sequence (Fig. 2). T2 plants were grown on MS plates and the segregation for the T-DNA bearing the WT POLD1 sequence was analyzed. As expected, about 3/4 of the plants displayed a WT leaf phenoype. Plants not segregating for the T-DNA displayed the curly leaf phenotype typical of gis5 mutants (arrows). (B) Multiple sequence alignment of the “finger B” from eukaryotic DNA polymerases. The green bar corresponds to the highly conserved distance between the K residue and the YG-pair. The K and YG-pair, in red, are fully conserved amino acids [71]. The arrow indicates the A residue mutated in gis5; #, essential aminoacids for DNA polymerization. Protein sequences were retrieved from SWISS-PROT (At, Arabidopsis thaliana; Sc, Saccharomyces cerevisiae; Os, Oriza sativa; Gm, Glycine max; Dd, Dictiostelyum discoideum; Rn, Rattus norvergicus; Mn, Mus musculus; Ma, Mesocricetus auratus; Bt, Bos taurus; Hs, Homo sapiens; Dm, Drosophila melanogaster; Ce, Caenorabditis elegans; Ca, Candida albicans; Sp, Schizosaccharomices pombe; Pf, Plasmodium falciparum. The program ClustalW2 was used for the alignment (https://www.ebi.ac.uk/Tools/msa/clustalw2/). (C) Substrate bound and substrate-free structural model of the catalytic subunit of DNA Polymerase d. The program “Modeller” [62] was used to construct the model using the X-ray structure of the yeast DNA Polymerase a catalytic subunit in its substrate-bound form (4FYD model,colored in light blue) and free form (4FVM model,colored in light brown). Note the displacement o the finger helix which contains Ala707 (arrows). (TIF) [file pgen.1004975.s001.tif]

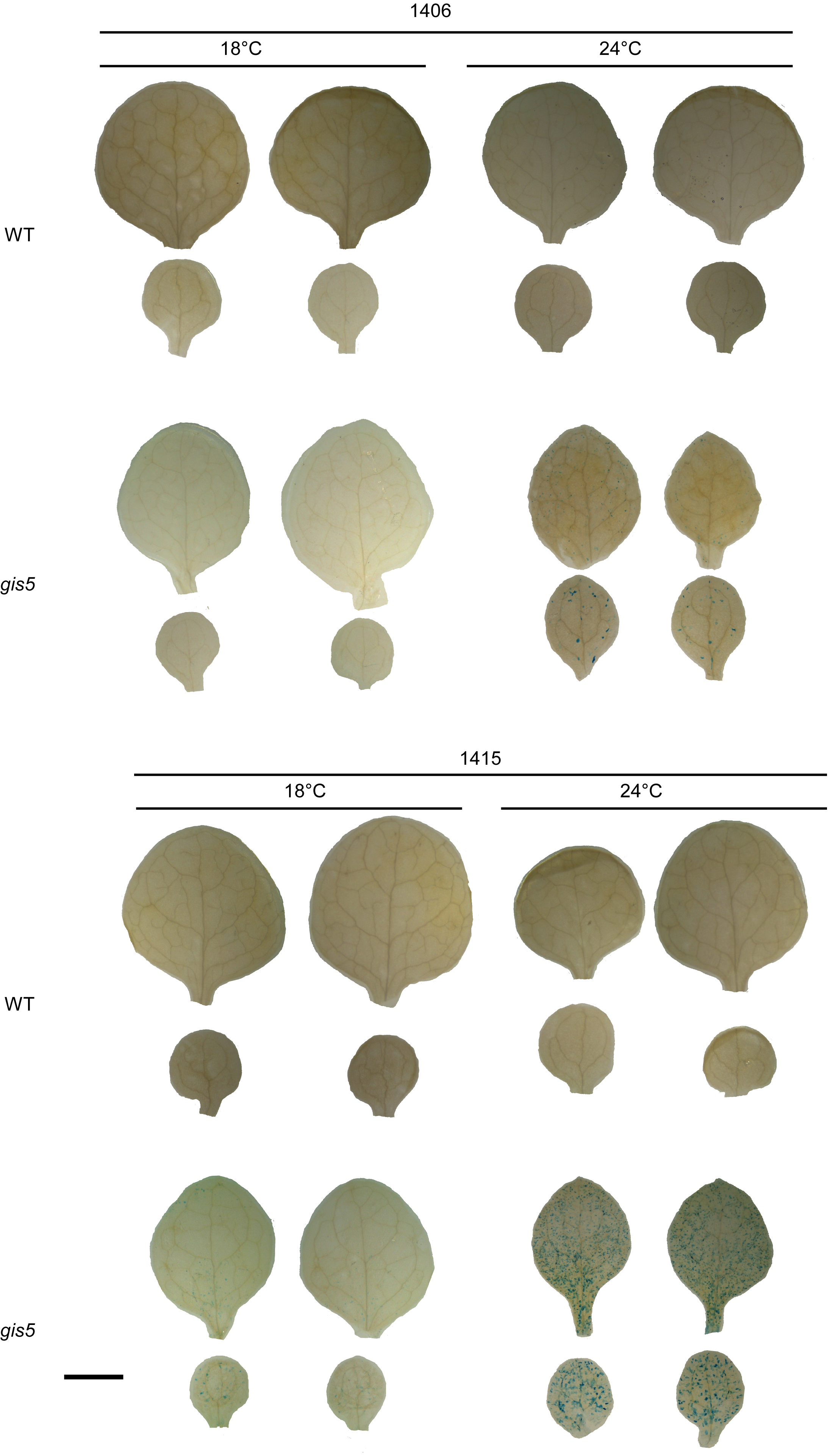

Supplement: S2 Fig — The gis5 mutants display increased HR in a temperature-dependent manner. gis5 mutant plants were crossed into HR reporter lines 1406 and 1415. WT and gis5 mutant plants bearing the 1406 or the 1415 reporters, as indicated, were grown in MS Agar plates under LD at either 18°C or 24°C. At bolting time, plant tissues were fixed and stained with X-Gluc. First leaves and cotyedons are shown. Dots indicate HR events which restored GUS activity [31]. Scale Bar: 1mm. (TIF) [file pgen.1004975.s002.tif]

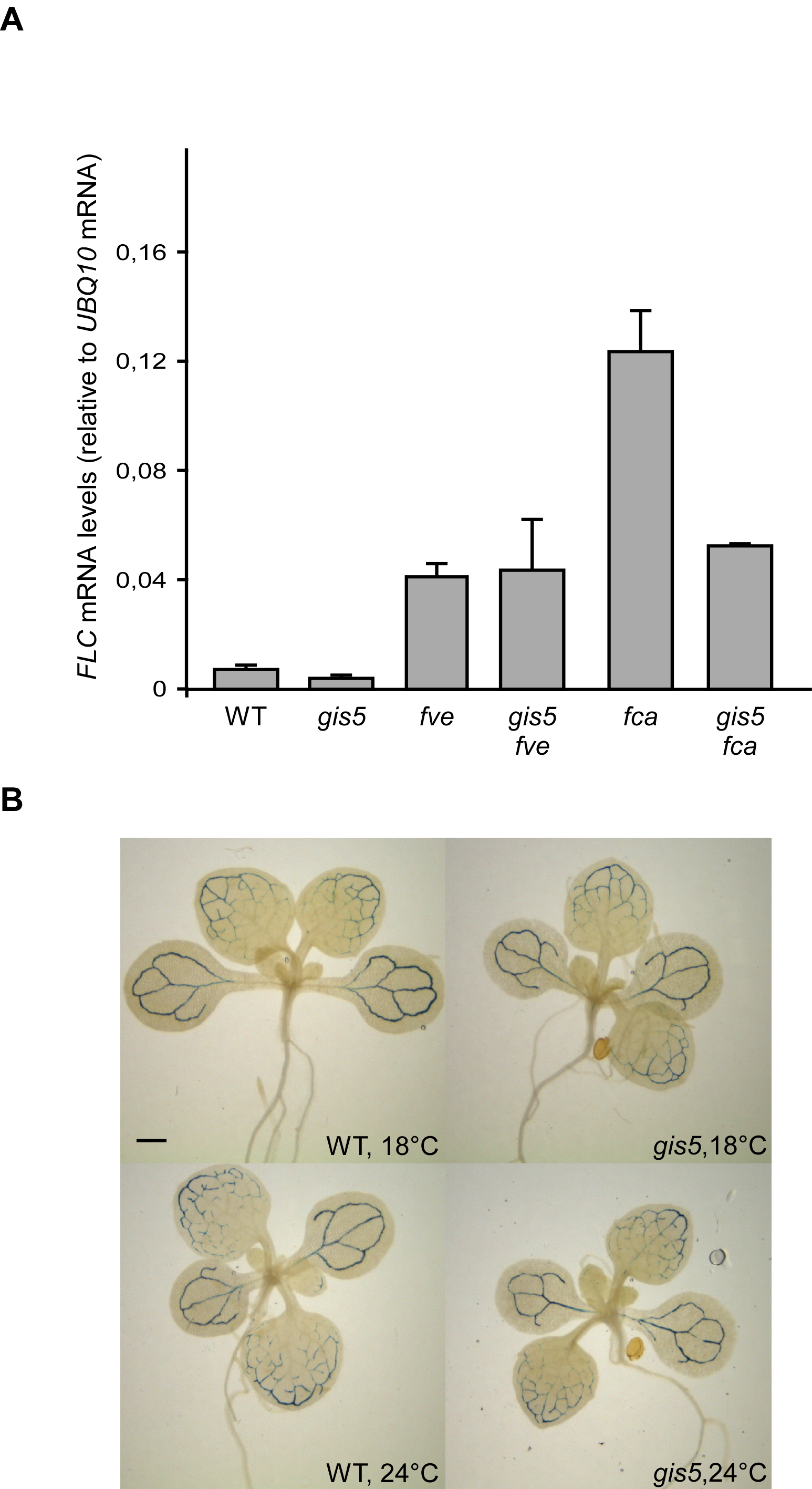

Supplement: S3 Fig — (A) The gis5 mutation affects FLC expression. WT, gis5, fve, fca, fve gis5 and fca gis5 mutant plants were grown for 10 days under continuous light at 24°C. Total RNA was extracted and quantitative Reverse Transcriptase-PCR (q-PCR) was performed as described in Materials and Methods to quantitate FLC mRNA expression relative to UBQ10 mRNA. Bars represent the mean ±SEM of 3 independent biological replicates, each replicate analyzed in triplicate. Note that fve and fve gis5 double mutants showed similar expression levels of FLC although they flower very differently (Fig. 3). (B) FT is expressed in vascular tissue in gis5 mutants. gis5 mutants were crossed into transgenic plants bearing P8.1kbFT:GUS. The 8.1 kb promoter fragment was shown to recapitulate endogenous FT expression patterns [6]. gis5 (right panels) and WT (left panels) plants homozygous for the reporter construct were grown in LD at the indicated temperatures. Plants were then fixed and tissue specific GUS activity revealed with X-Guc as a substrate. (TIF) [file pgen.1004975.s003.tif]

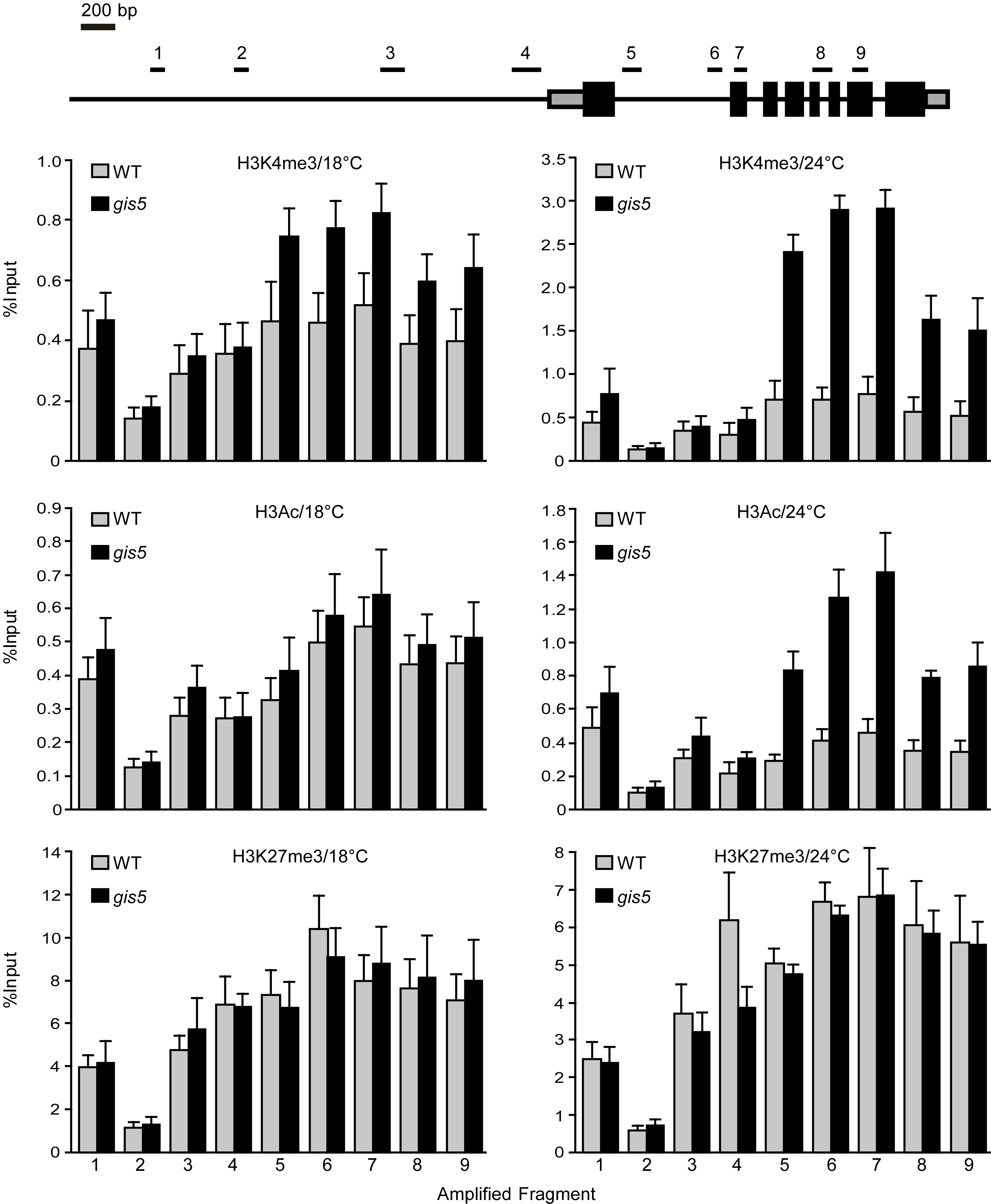

Supplement: S4 Fig — WT and gis5 mutant plants were grown for 10 days under continuous light at either 18°C or 24°C. Enrichment in H3K4me3, H3Ac and H3K27me3 was determined by ChIP followed by qRT-PCR of the fragments depicted in the top panel. Bars represent the mean ± SEM of 5–6 independent biological replicates. (TIF) [file pgen.1004975.s004.tif]

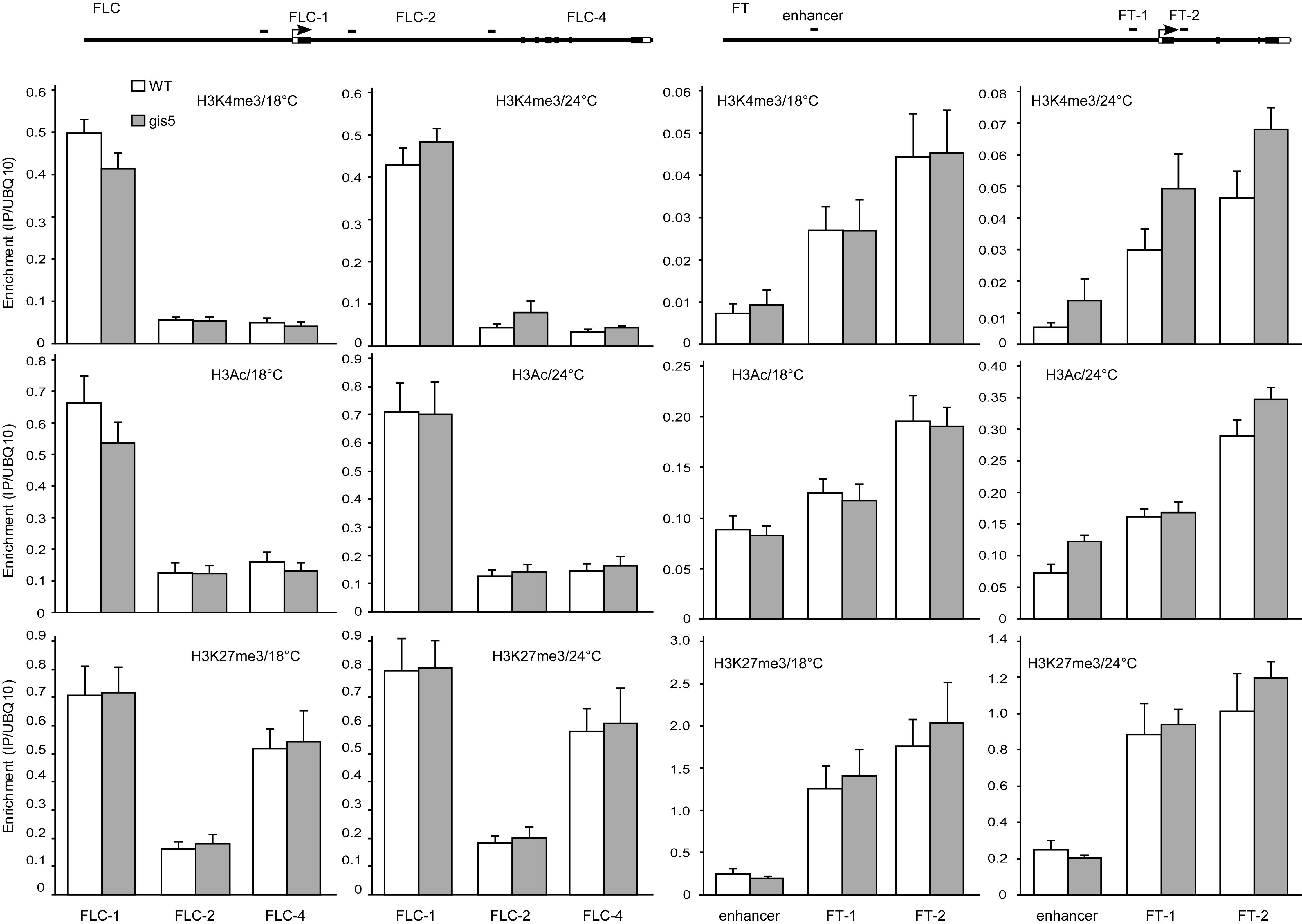

Supplement: S5 Fig — WT and gis5 mutant plants were grown for 10 days under continuous light at either 18°C or 24°C. Enrichment in H3K4me3, H3Ac and H3K27me3 was determined by ChIp followed by q-PCR of the fragments depicted in the top panel. Data was relativized to a UBQ10 fragment. Bars represent the mean ±SEM of 5–6 independent biological replicates. (TIF) [file pgen.1004975.s005.tif]

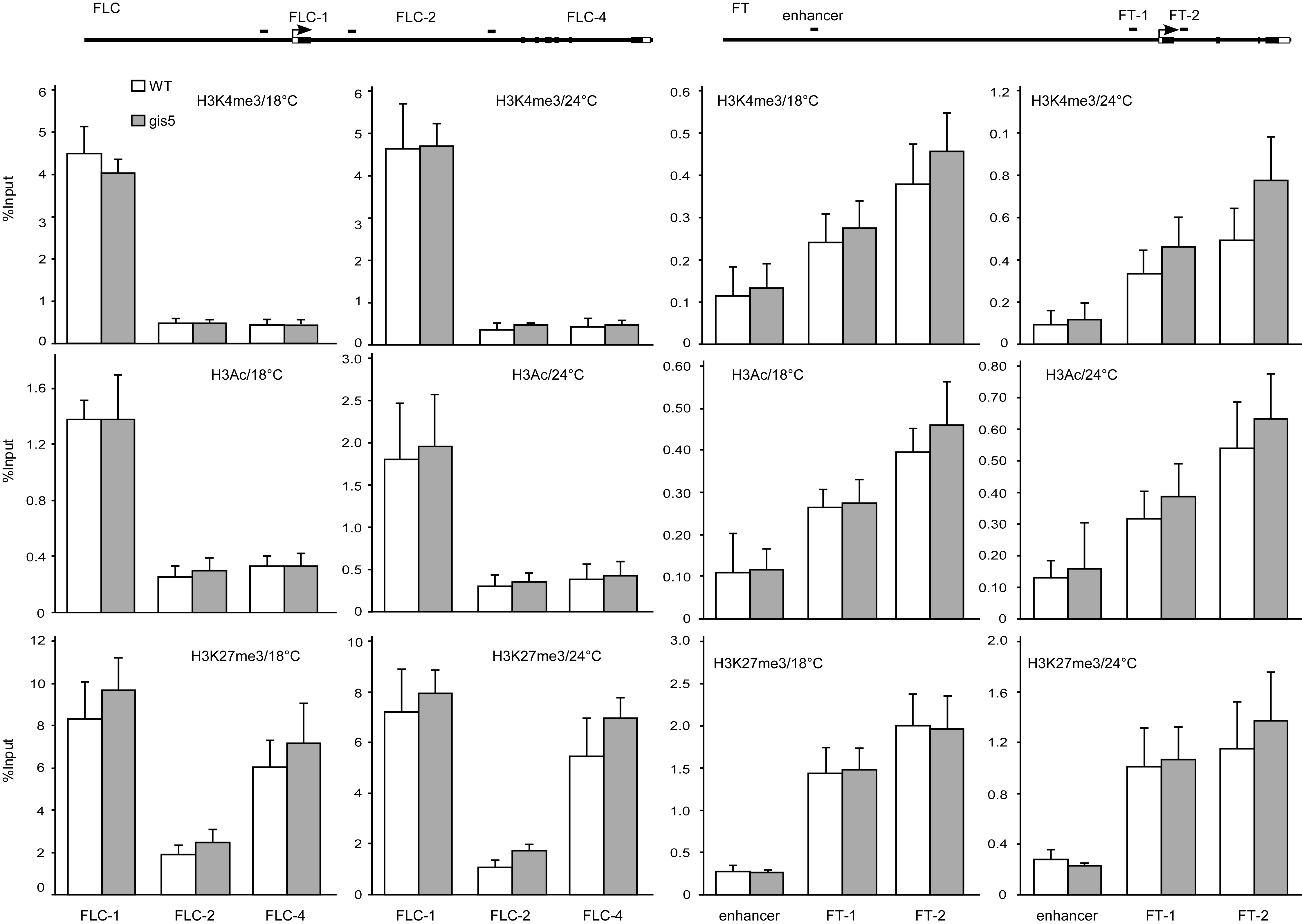

Supplement: S6 Fig — Bars represent the mean ±SEM of 5–6 independent biological replicates. (TIF) [file pgen.1004975.s006.tif]

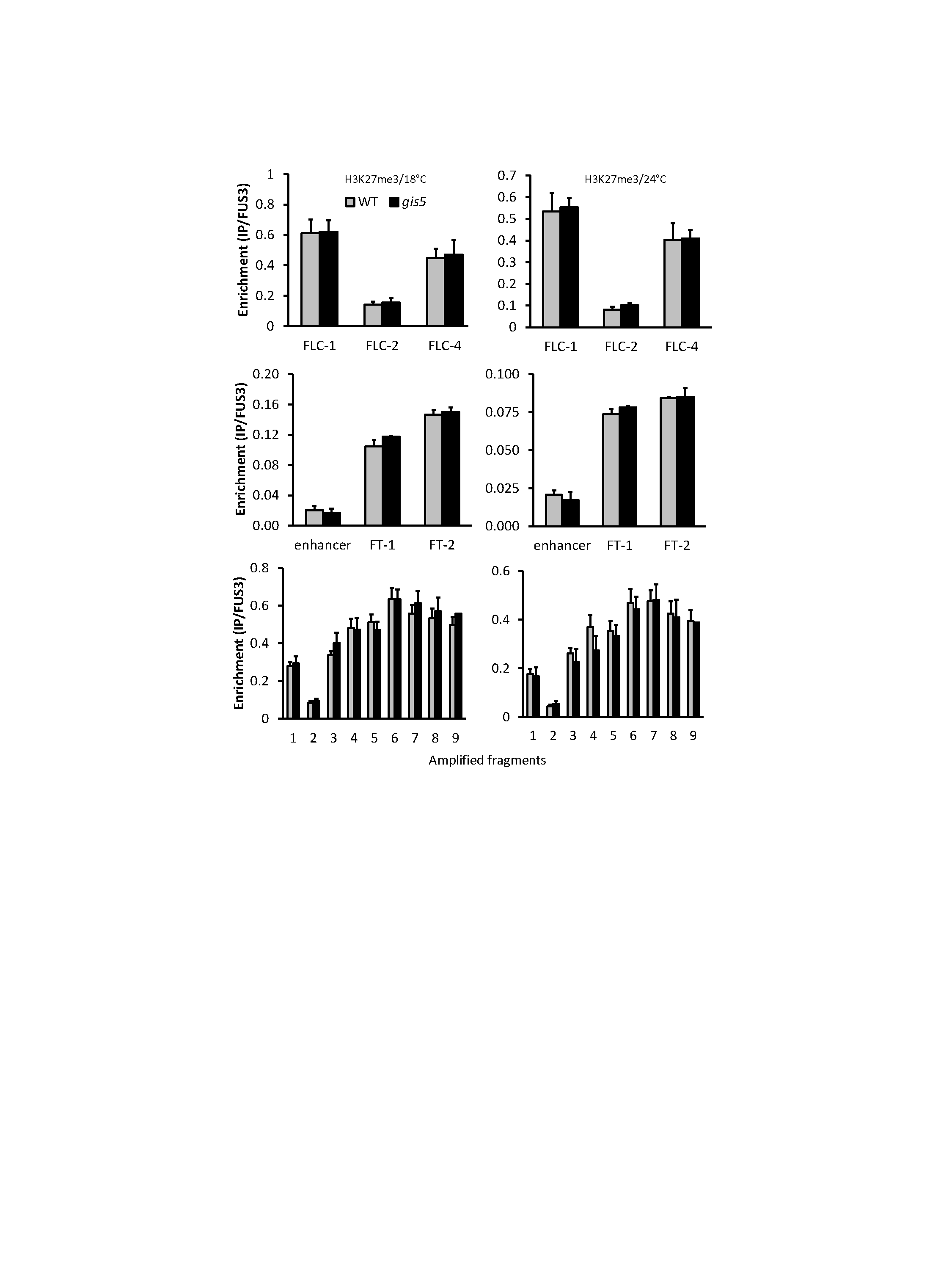

Supplement: S7 Fig — (TIF) [file pgen.1004975.s007.tif]

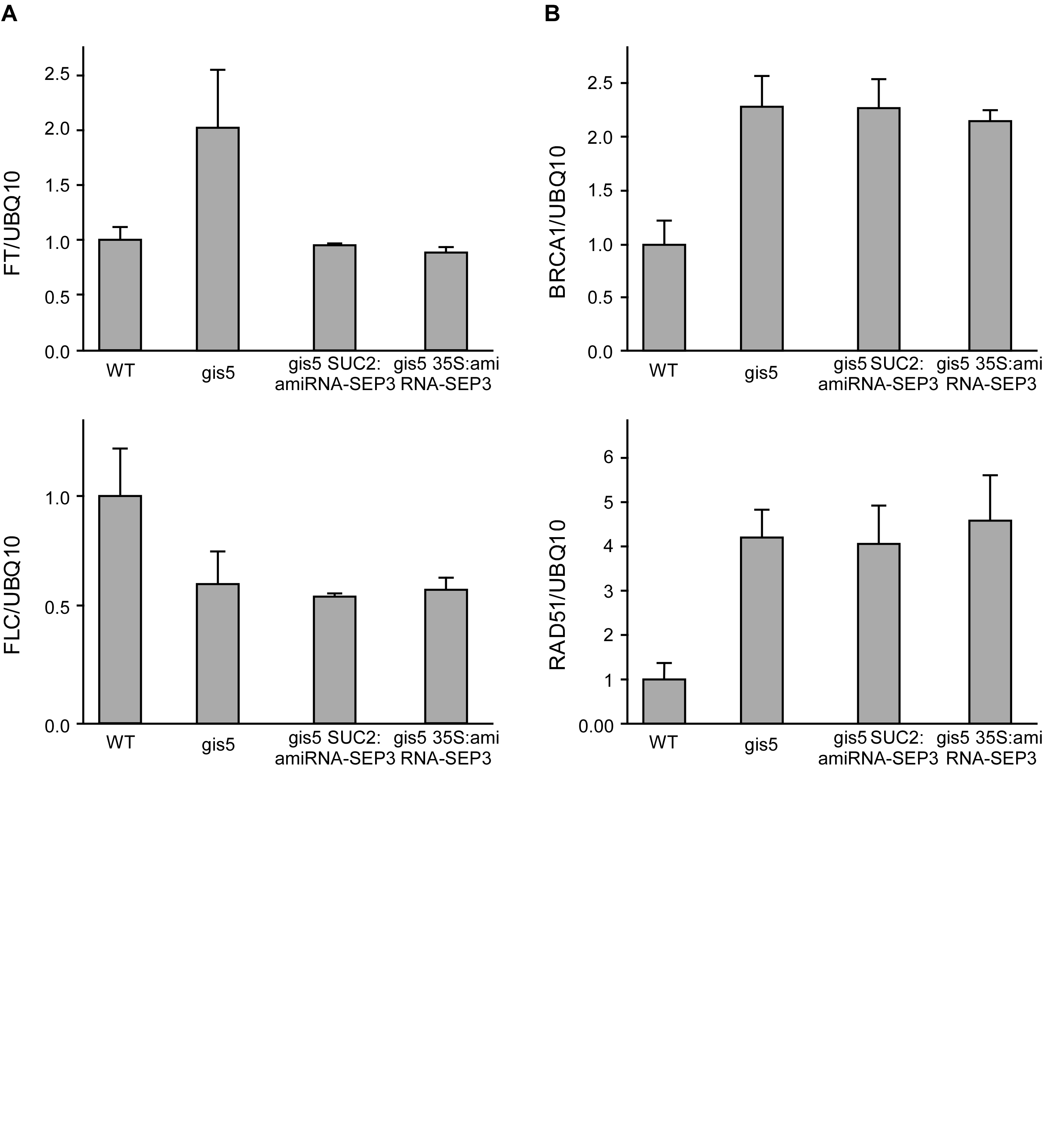

Supplement: S8 Fig — (A) SEP3 overexpression in phloem tissue is required for FT expression but not for FLC expression. Plants of the genotypes indicated on the abscissa were grown for 10 days under continuous light at 23°C. Total RNA was extracted and quantitative Reverse Transcriptase-PCR (q-PCR) was performed as described in Materials and Methods to quantitate FT (top panel) and FLC (lower panel). (B) The DNA replication stress response does not depend on SEP3 overexpression. mRNAs for BRCA1 (top panel) and RAD51 (lower panel) genes were quantified as above. In each panel, WT mRNA were scaled to one. Bars represent the mean ±SEM of 3 independent biological replicates, each replicate analyzed in triplicate. (TIF) [file pgen.1004975.s008.tif]

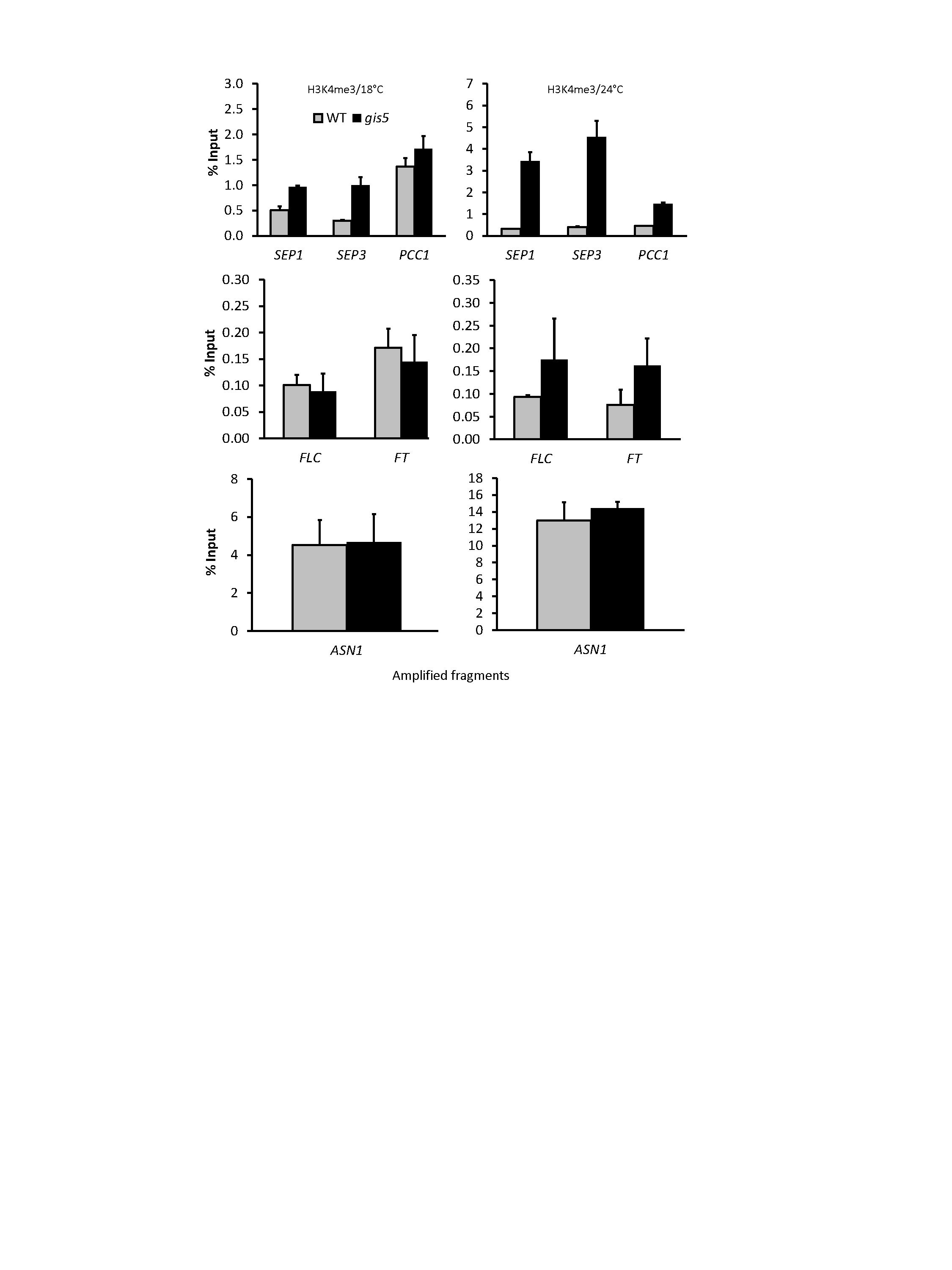

Supplement: S9 Fig — WT and gis5 mutant plants were grown for 10 days under continuous light at either 18°C or 24°C. Enrichment in H3K4me3 was determined by ChIP followed by qRT-PCR at the loci indicated in the abcissas. Data were expressed as a fraction of input. Bars represent the mean ± SEM of 3 independent biological replicates. (TIF) [file pgen.1004975.s009.tif]
