# Supplementary material for: The Arabidopsis DNA Polymerase δ Has a Role in the Deposition of Transcriptionally Active Epigenetic Marks, Development and Flowering
Source: PLoS Genet. 2015 Feb 18;11(2):e1004975. doi: 10.1371/journal.pgen.1004975 (PMC4334202; doi:10.1371/journal.pgen.1004975)
Supplement: S1 Text — (PDF) [file pgen.1004975.s010.pdf]

Primers Sequences

Primer Sequences for Genotyping Insertional Mutants

ft-10 (At1g65480, GABI_290E08)

ft-10-1: GATGAATCTCTGTTGTGGAATATTTGAAAACTG

ft-10-2: CTAGGACTTGGAACATCTGGATCCACCA

GABI RB2: TTTCTCCATATTGACCATCATACTCATTGC

soc1-2 (At2g45660, SALK_006054)

soc1-2-1: ATCGAGTCAGCACCAAACCGGTTTCTGAA

soc1-2-2: CAACGAAAGATTAAGTACCCAAAAAGCAA

LB1: GGCAATCAGCTGTTGCCCGTCTCACTGGTG

flc-3 (At5g10140, SALK_003346)

flc-3-1: CCTAGAGGCACCAAAGAAACAAGGCTGTGT

flc-3-2: CTCTTGGATTTGTATATGCACGTCCGGGAG

LB1: GGCAATCAGCTGTTGCCCGTCTCACTGGTG

co-9 (At5g15840, SAIL_24_HO4)

co-9-1: GTAGCTCGTCTGTGGTACGCTGCAGTTT

co-9-2: GATGATGCCTCTCTATGCACAGCCTGT

LB3: TAGCATCTGAATTTCATAACCAATCTCGATACAC

tsf-3 (At4g20370, SALK_087522)

tsf-3-1: GTGGCAGGTATATCAGTCACCAACCTGTA

tsf-3-2: TGTGTGTGTCATGTTATATCATAGCTTGCA

LB1: GGCAATCAGCTGTTGCCCGTCTCACTGGTG

gi-2 (At1g22770, CS3397)

gi-2-1: TGCTGCTCAGTTTTATAAATGGGACGGTT

gi-2-2: GTGTACAGCAAGGAAGCTCATCCGTT

gi-2-3: CAATACATAGACCTCAGCAGAGAGACCAAA

gi-2-4: AGAAATCCTTCGCATTTTGACTCATTACAATT

Primer Sequences for Mapping

CER457684

GGGAATTCTCAGATTCTTGGTATGGTAGATA

ATCAAAAGAGACAGCTTCAGAAGTTTATGC

CER457265

AATTTTTGCCTAGTTCAGTGATTGCTCAA

CAAGGCTATTATCACTTCCGAAGAGGTTAGTA

CER457069

TGTTGTGGTTGTGTCCAGCTCCAT

TCCAAAACAACCGGTTTTGATAAGGAT

CER437610

GGTTTACAACAACTTGAAGATTTGTGATATTAATTT

GATCCATACTCTTATTGTTATTCTAATGAATCATGATA

CER436454

GGAACATCTATACTACTATGAAGAGCTGAAAAG

GCTTCTCTTGGGAGAAACTTCAGTTTCAA

CER456030

TTACTGAAGCTGGGGCTATTGCAGATA

GAGTATGTGACGATACCTCTTTTTTCATGATCTA

CER456203

TCTCTGTACATTTCAACTCACATAGCATTGAA

TTCCATGGTTCTAGAACCGCTTAAGCTA

CER455192

CCATGCTTTTGAAAAGCACCAAATTTA

CGACCCGAATACGAAAGTCAAGTCAA

CER436434

GCCTTTTGCTTTTCACCTTTCGTTATAA

GTGAGAATCGGAATCGAATTCACCA

CER456709

CGCCATTATCTTGTATAATATGCATGGTGTA

AGAAAATTATGCAAAATATTATGATCTTGCCAT

CER454467

CATTTCCTCCATTAATCCTTAACGAGACAA

TCCTTGTTTTGCTTCTTCTCTCCTTGAA

CER465586

TGAGTTTTTTACAAAGGCCAAGTTTGTTTCT

TGAAAATGGAATTATCGGAAATGACGATT

CER448484

TGTGCATGGTATTATAGGTGGACCAGATTAT

GGTGGACATTTGTATCTTTTCGGTTTCTT

CER464657

CCTTGAGGTGGGCCATAACAAGCTAT

CACGACCCCTGTGTACCCATTCCTA

CER458022

TCCCTCATTCTTATAGCAAAGCTTTCGAT

CGAAGTGCAGTTGGGAGTCTCATGTA

CER460945

GGTCATCCTTGCAAGATATCAAAAGTTTGT

TGACAATTTTCTTCTATATAAGGATGTGGAGAAA

CER449342

TGGGTTCTTAAATATTTGCGGCTGAA

GCTCACCCTGAAACTCTCCTCCAGTT

CER448446

ATCGACTTAGGAAGGTCAGGGTCTGAA

ATTTCAAAACTCCCATCTTGCTTCAACTT

CER460835

CGCCGTCAATCTCTCCAAAATAAGAA

CAAATACATAGCCCTAAAGCGTGTCGAT

CER461379

ATGCAGTCGAGAGCTGTCTCAGGCT

GTGCTTGACGGTTTGTCGACGAA

CER459323

CGATTAAACTCAATCCAACGGCTTAGAGATA

CAAGACTTTGCAAACATTTACTAATGAACGA

CER459358

ACCAACGATCCCCTTTTGA

AACTGGTTGGTTTAAGAATAA

CER458362

CGTGTTGGCCTAAATCAGAGAGAGCAT

TGCCAGAGAAACCAAGAAATTGATACAATTA

CER464712

CAATAAGAAGAAGGAAGCGTACTGCACA

GTGAATGAACACATGGTGTGTTGTAACATTA

CER456185

CGGTAAATCTCTGCTTATTCTATTCACTGCAT

TCCAGTATTCATAAGCTTTTATGCTCCAAAATA

CER460458

GTTTGTGACGAATAGTGAAAGGAGAGAAGTAA

GAGAACCAACATAAATCCGTTACAATGTCAA

CER460130

CACAACACATCTTAGCTATTGTTTCAAGATCAA

GCATGCCTTAACTACATTCCTCACAGGTA

CER450937

GTCTCTAATGGAGAGACTTGGTTATAGAAGTTCAT

GGACAATAATCATTGCAAAGCTACCATCTATA

CER449332

CCCACTGCTTGGACAGTGTACGAA

GAATCCGATGGATAGAGGAAAAATGAATGTA

CER459891

TAACTTTCCTTTTCCCTACCAAATTCCAA

CAACACTCTACCCCACTACTTTTATTTTGGTT

CER466221

ATCAAACGCCAACTGAATCAATCAATTAA

GTGTGTGGACTCACCATTGGTCACAT

CER451656

AAAAGACAGAGATGATCATTAAGCACATTCAA

GATTTGACAATCACGGATACAAAAGCTGT

CER449857

CCTGGCGCTAATGTACACTCTTCGTT

TGTAATCTATCTCTCACTCACGATGCCTCTT

CER456519

ATGGCTGAAAAGGTTGTACAATAGATGTTATAATT

GGGAAACACAATTAAATGGTGCTGCA

CER457559

ACATTGCTTTGAGACGCATAGCCATT

CAACAAGTCACAACGTTGGTAACAAACC

K9D7

TGTGATAGGGTTTATGTTCGTGTCAATGA

ATCGAGGCATATGCCACGTTTCTTA

K15N18

CACTTCAGGTTCGATCATCTTCAACATC

AATCTTTGCTATAATTCGGTTGGTCAGCTA

CER456386

AGTAACTCAAAACCAAACAAGGAC

TTTGATGCATCATAACAGAAAGTC

Primer Sequences for q-PCR

BRCA1

BRCA1-1: GCGAGGAGCCATATGAGATCACT

BRCA1-2: CCT CCTGCTGCTACAATCAGATCT

FLC

FLC-1: CTCCTCCGGCGATAACCTGGT

FLC-2: CATGATGATTATTCTCCATCTGGCT

FT

FT-1: CTGGAACAACCTTTGGCAAT

FT-2: TACACTGTTTGCCTGCCAAG

RAD51

RAD51-1: CCTTTCCTCAACGCCAACCTTGTT

RAD51-2: GGAGAGCTTTCGGCTCGACAAAT

SEP1

SEP1-1: GGTTGTCGCAGTTATTTGCTCTGAG

SEP1-2: GAGCTTTGGCAATGAAGCTGGAT

SEP2

SEP2-1: CGTCACAGCCATTTGCTCTGAG

SEP2-2: AGAGCTTTGTCAATGAAGCTGGAAG

SEP3

SEP3-1: CATCTTGTTGCCCCTGATACCCGAT

SEP3-2: GCTTGACCAGCTCAACGATCT TCAG

UBQ10

UBQ10-1: TCAATTCTCTCTACCGTGATCAAGATGCA

UBQ10-2: GGTGTCAGAACTCTCCACCTCAAGAGTA

Primers for ChIP assays

FT

FT-enhancer (distal promoter)-1: GGCCAACATTAGAAGAAGATTCC

FT-enhancer (distal promoter)-2: TCTTGACATGGAGCGAAAGA

FT-1-1 (proximal promoter): GGGTTTGGAATACCACAAACAGA

FT-1-2 (proximal promoter): ACTCGGGTCGGTGAAATCAT

FT-2-1 (proximal intron 1): TGGTGGAGAAGACCTCAGGAACTT

FT-2-2 (proximal intron 1): CGTGGGGCATTTTTAACCAAGGTC

SEP3

SEP3-1-1 (distal promoter): CACCGTTTCACGCCAAAGTTCGT

SEP3-1-2 (distal promoter): CTCGCCCCGGCCCAAAACAT

SEP3-2-1 (promoter): TCGGACGGCTTTGAGGCAATGT

SEP3-2-2 (promoter): TGACGTGGACCCTGTCCGTCT

SEP3-3-1 (promoter): GACTTTCGGTCAGACATGATCTC

SEP3-3-2 (promoter): GGTAAGAGAGTCTGATGAGACAC

SEP3-4-1 (proximal promoter): GTGTTGGTGAGAGTGGAACTC

SEP3-4-2 (proximal promoter): ACTCTCAGACTCAACTATATACCC

SEP3-5-1 (proximal intron 1): GTCTTGTATGTATGGGTCTCTC

SEP3-5-2 (proximal intron 1): ATGATACAGATTTGGGGTATCC

SEP3-6-1 (distal intron 1): GGATATTGTTTCCACGACAATCC

SEP3-6-2 (distal intron 1): AGATGAATTTGACATTAGCGTCA

SEP3-7-1 (exon 2): GGCCTCTCTTGAAGGCACATTGGG

SEP3-7-2 (exon 2): GCATGCTTCGGACACTGGAGAGG

SEP3-8-1 (exon 3): CTCAGTCAGCATGCGTTCCTGC

SEP3-8-2 (exon 3): GCTTGACCAGCTCAACGATCTTCA

SEP3-9-1 (exon 4): GGCTGGAAGAAAGCTTGGGAGTGT

SEP3-9-2 (exon 4): AGATGCCACTCCAGCTGAACCCT

FLC

FLC-1-1 (promoter): CCCAGGTAAGGAAAAGGCGT

FLC-1-2 (promoter): ACCTTCTCCAAACGTCGCAA

FLC-2-1 (proximal intron 1): ACGTGTACCGCATGACGAG

FLC-2-2 (proximal intron 1): TCCGTATCGTAGGGGAGGAAA

FLC-3-1 (distal intron 1): AAATCTCCCGGACGTGCATA

FLC-3-2 (distal intron 1): TGAATCACAATCGTCGTGTGC

UBQ10

UBQ10-1: TCCAGGACAAGGAAGGTATTCCTCCG

UBQ10-2: CCACCAAAGTTTTACATGAAACGAA

ASN1

ASN1-1 (first intron): AGAGGTTGATCACCGGAAGC

ASN1-2 (first intron): CAGAGGACCTGACTGGAGTG

SEP1

SEP1-1 (first intron): GTTGTTTACTTTGAGGAGCTGCAA

SEP1-2 (first intron): ATTGTCTGTTCTCTGTGATGCTGA

FUS3

FUS3-1 (first intron): AGTTGGCACGTGGGAAATAG

FUS3-2 (first intron): GTGGCAAGTGTTGATCATGG

PCC1

PCC1-1 (first intron):TTGGAGTTTGTCTCCACTGCT

PCC1-2 (first intron): CTTCATCAGGGCCGTACACA

Primers used for artificial miRNA constructs

SEP1

I miR-s: gaTATACCGGATTATCATACCGCtctctcttttgtattcc

II miR-a: gaGCGGTATGATAATCCGGTATAtcaaagagaatcaatga

III miR*s: gaGCAGTATGATAATGCGGTATTtcacaggtcgtgatatg

IV miR*a: gaAATACCGCATTATCATACTGCtctacatatatattcct

SEP3

I miR-s: gaTAAACTCGTACAGCGTTCCTCtctctcttttgtattcc

II miR-a: gaGAGGAACGCTGTACGAGTTTAtcaaagagaatcaatga

III miR*s: gaGAAGAACGCTGTAGGAGTTTTtcacaggtcgtgatatg

IV miR*a: gaAAAACTCCTACAGCGTTCTTCtctacatatatattcct
