# Supplementary material for: Correction: The Arabidopsis DNA Polymerase δ Has a Role in the Deposition of Transcriptionally Active Epigenetic Marks, Development and Flowering
Source: PLoS Genet. 2015 Jun 15;11(6):e1005318. doi: 10.1371/journal.pgen.1005318 (PMC4468264; doi:10.1371/journal.pgen.1005318)
Supplement: S2 File — (PDF) [file pgen.1005318.s002.pdf]

PLOS | GENETICS

RESEARCH ARTICLE

The *Arabidopsis* DNA Polymerase δ Has a Role in the Deposition of Transcriptionally Active Epigenetic Marks, Development and Flowering

Francisco M. Iglesias[1], Natalia A. Bruera[1], Sebastián Dergan-Dylon[1], Cristina Marino-Buslje[1], Hernán Lorenzi[2], Julieta L. Mateos[1,3], Franziska Turck[3], George Coupland[3], Pablo D. Cerdán[1,4]*

1 Fundación Instituto Leloir, IIBBA-CONICET, Buenos Aires, Argentina, **2** J. Craig Venter Institute, Rockville, Maryland, United States of America, **3** Max Planck Institute for Plant Breeding Research, Cologne, Germany, **4** Facultad de Ciencias Exactas y Naturales, Universidad de Buenos Aires, Buenos Aires, Argentina

* pcerdan@leloir.org.ar

CrossMark
click for updates

OPEN ACCESS

Citation: Iglesias FM, Bruera NA, Dergan-Dylon S, Marino-Buslje C, Lorenzi H, Mateos JL, et al. (2015) The *Arabidopsis* DNA Polymerase δ Has a Role in the Deposition of Transcriptionally Active Epigenetic Marks, Development and Flowering. PLoS Genet 11 (2): e1004975. doi:10.1371/journal.pgen.1004975

Editor: Gregory P. Copenhaver, The University of North Carolina at Chapel Hill, UNITED STATES

Received: August 29, 2014

Accepted: December 29, 2014

Published: February 18, 2015

Copyright: © 2015 Iglesias et al. This is an open access article distributed under the terms of the Creative Commons Attribution License, which permits unrestricted use, distribution, and reproduction in any medium, provided the original author and source are credited.

Data Availability Statement: All relevant data are within the paper and its Supporting Information files, except for the microarray data. All microarray data files are available from the GEO database (accession number GSE58036).

Funding: This work was supported by grants PICT2011-1610 from the AGENCIA NACIONAL DE PROMOCIÓN CIENTÍFICA Y TÉCNICA(ANPCyT, www.agencia.mincyt.gob.ar) to PDC. FMI was supported by a fellowship from CONICET, CONSEJO NACIONAL DE INVESTIGACIONES CIENTÍFICAS Y TÉCNICAS (CONICET, www.conicet.gov.ar/)and the

Abstract

DNA replication is a key process in living organisms. DNA polymerase α (Polα) initiates strand synthesis, which is performed by Polε and Polδ in leading and lagging strands, respectively. Whereas loss of DNA polymerase activity is incompatible with life, viable mutants of Polα and Polε were isolated, allowing the identification of their functions beyond DNA replication. In contrast, no viable mutants in the Polδ polymerase-domain were reported in multicellular organisms. Here we identify such a mutant which is also thermosensitive. Mutant plants were unable to complete development at 28°C, looked normal at 18°C, but displayed increased expression of DNA replication-stress marker genes, homologous recombination and lysine 4 histone 3 trimethylation at the *SEPALLATA3* (*SEP3*) locus at 24°C, which correlated with ectopic expression of *SEP3*. Surprisingly, high expression of SEP3 in vascular tissue promoted *FLOWERING LOCUS T* (*FT*) expression, forming a positive feedback loop with *SEP3* and leading to early flowering and curly leaves phenotypes. These results strongly suggest that the DNA polymerase δ is required for the proper establishment of transcriptionally active epigenetic marks and that its failure might affect development by affecting the epigenetic control of master genes.

Author Summary

Three DNA polymerases replicate DNA in Eukaryotes. DNA polymerase α (Polα) initiates strand synthesis, which is performed by Polε and Polδ in leading and lagging strands, respectively. Not only the information encoded in the DNA, but also the inheritance of chromatin states is essential during development. Loss of function mutants in DNA polymerases lead to lethal phenotypes. Hence, hypomorphic alleles are necessary to study their

Deutscher Akademischer Austauschdienst (DAAD, https://www.daad.de/) Short Term Fellowship Programme. The funders had no role in study design, data collection and analysis, decision to publish, or preparation of the manuscript.

Competing Interests: The authors have declared that no competing interests exist.

roles beyond DNA replication. Here we identify a thermosensitive mutant of the Polδ in the model plant *Arabidopsis thaliana*, which bears an aminoacid substitution in the polymerase-domain. The mutants were essentially normal at 18°C but arrested development at 28°C. Interestingly, at 24°C we were able to study the roles of Polδ in epigenetic inheritance and plant development. We observed a tight connection between DNA replication stress and an increase the deposition of transcriptionally active chromatin marks in the *SEPALLATA3* (*SEP3*) locus. Finally, we tested by genetic means that the ectopic expression of *SEP3* was indeed the cause of early flowering and the leaf phenotypes by promoting the expression of *FLOWERING LOCUS T* (*FT*). These results link Polδ activity to the proper establishment of transcriptionally active epigenetic marks, which then impact the development of multicellular organisms.

Introduction

Arabidopsis is a facultative long-day (LD) plant, meaning that LDs accelerate flowering whereas in short days (SD) flowering is delayed. Flowering in spring (LDs) is therefore promoted by *GIGANTEA* (*GI*), *CONSTANS* (*CO*) and *FLOWERING LOCUS T* (*FT*) which constitute the so called "photoperiod pathway". GI activates CO which eventually accumulates in the late afternoon and early night under LD conditions and induces the expression of *FT* in phloem companion cells. The protein FT is an universal florigen and moves to the apical meristem to promote the transition to flowering [1–3].

In plants, epigenetic inheritance confers a cellular memory of past environmental conditions. The winter ecotypes of *Arabidopsis thaliana* require a prolonged exposure to near freezing temperatures, or vernalization, to become competent to flowering in spring [4]. Plants are able to "remember" the past winter because persistent cold exposure (2–4 weeks) activates an epigenetic mechanism that requires the trimethylation of Lysine 27 of histone 3 (H3K27me3), which permanently represses *FLOWERING LOCUS C* (*FLC*) [4].

The pathways that respond to photoperiod and vernalization are integrated at the level of *FT*, which is directly repressed by FLC [4,5]. The *FT* gene is also known as a "flowering integrator" because it also responds to other flowering pathways including the thermosensory and autonomous pathways [4,5]. The complex regulation of *FT* expression is due at least in part to epigenetic mechanisms [6–10]. The chromatin of *FT* is enriched in H3K27me3; *curly leaf* (*clf*) and *terminal flowering 2/like heterochromatin protein 1* (*tfl2/lhp1*) mutants, which are defective in the H3K27 methylase and H3K27me3 binding activity, are early flowering and photoperiod insensitive due to high expression of *FT* [9,11–13].

The terminal differentiation of specialized tissues in multicellular organisms is strongly influenced by the previous DNA replication events of the cells that constitute each tissue. In Eukaryotes, three replicative DNA polymerases (Polα, Polε and Polδ) are responsible for the faithful duplication of the nuclear genome [14]. Polα forms a complex with a primase to initiate replication at origins and Okazaki fragments and these short stretches of RNA-DNA hybrids are extended by Polε in the leading strand and Polδ in the lagging strand [14].

Apart from their essential role in DNA replication, Polα and Polε are required for other non-essential functions such as the maintenance of transcriptional silencing in yeast. In this alternative role, Polε is part of a mechanism that processes transcripts into siRNAs to reinstall transcriptional silencing [15]. In plants, mutants versions of Polα and Polε led to changes in histone marks, resulting in elevated *FT* expression together with increased flower homeotic gene expression, which produced early flowering and curly leaf phenotypes [11,16–19].

However, which genes are the primary targets of the defective-polymerase induced epigenetic changes remains unclear.

Unlike Polα and Polε, the role of the Polδ in epigenetic inheritance has not been addressed so far. Besides lagging strand synthesis, Polδ also participates in many other processes that repair DNA lesions required to protect genome integrity [20,21].

Reports in yeast and plants have shown that a reduction in the amount of Polδ leads to genome instability and hyperrecombination phenotypes [20–22]. In mammals, mutations in the proofreading domain of Polδ produce predisposition to cancer. For instance, a POLD1 S478N variant in human populations predisposes to colorectal tumors and endometrial cancer [23].

Although the fact that replicative polymerases are essential in most studied organisms, viable mutant alleles of *POLA1* (At5g67100, encoding the catalytic subunit of Polα and *POLE1* (At1g08260, encoding the catalytic subunit of Polε were isolated in multiple genetic screenings [11,16,18,19,24,25]. However, viable hypomorphic alleles of *POLD1* were not isolated so far, which may be due to its essential roles in DNA replication and repair. Here, we report the isolation of *gigantea suppressor 5* (*gis5*), the first plant *POLD1* viable mutant allele, which also proved to be thermosensitive. Under restrictive temperatures, the *gis5* allele led to early flowering and curly leaf phenotypes which were dependent on the *FT* gene but caused by overexpression of *SEP3*, which showed a correlation with increased trimethylation of Lysine 4 (H3K4me3) at the *SEP3* locus. These phenotypes mostly disappeared at permissive temperatures. Our findings reveal an unforeseen function of Polδ that may be linked to the correct establishment of transcriptionally active epigenetic marks during DNA replication.

Results

Isolation of *gigantea suppressor 5*

We performed a genetic screen for induced mutations that suppressed the late flowering phenotype of *gi-2* mutants, with the aim to isolate genes involved in the interaction between photoperiod and thermosensory pathways. One of the ethyl methanesulfonate (EMS)-induced mutations suppressed most of the *gi-2* late flowering phenotype under long days (LD) conditions and was named *gigantea suppressor 5* (*gis5*) (Fig. 1A). The *gis5* mutation also accelerated flowering in a wild type (WT) background (Fig. 1A) so we used *gis5* in this background in subsequent experiments. We also observed that in the WT background—but not in the *gi-2* background–, *gis5* displayed a curly leaf phenotype, reminiscent of *curly leaf* (*clf*) mutants [12]. Interestingly, the curly leaf phenotype depended on temperature, i.e.: it was strong at 24°C but disappeared at 18°C (Fig. 1B-C). We decided to evaluate whether the flowering phenotype of *gis5* mutants was also temperature-dependent. Under LD, the flowering phenotype of *gis5* mutants was relatively insensitive to temperature in the range 18–24°C (Fig. 1D). In stark contrast, the *gis5* early flowering phenotype was mostly suppressed when plants were grown at 18°C under short days (SD) (Fig. 1E).

gis5 Encodes the Catalytic Subunit of the DNA Polymerase δ

We mapped *gis5* to a 120kb interval delimited by markers CER436434 and CER436454 (http://www.arabidopsis.org/browse/Cereon/index.jsp [26]) (Fig. 2A). We detected a C to T transition in the 18th exon of the gene encoding the catalytic subunit of Polδ (*POLD1*, AT5g63960) which led to a A707V substitution (Fig. 2A). To confirm that the *gis5* mutation was the cause of the observed phenotypes, we complemented *gis5* mutants with a WT genomic fragment containing the complete *POLD1* gene with its own promoter and terminator sequences. Four independent transgenic lines, with single *locus* T-DNA insertions were evaluated and in all cases, the curly leaf phenotypes and the early flowering under both LD and SD were complemented to a high degree (Fig. 2B; S1A Fig.).

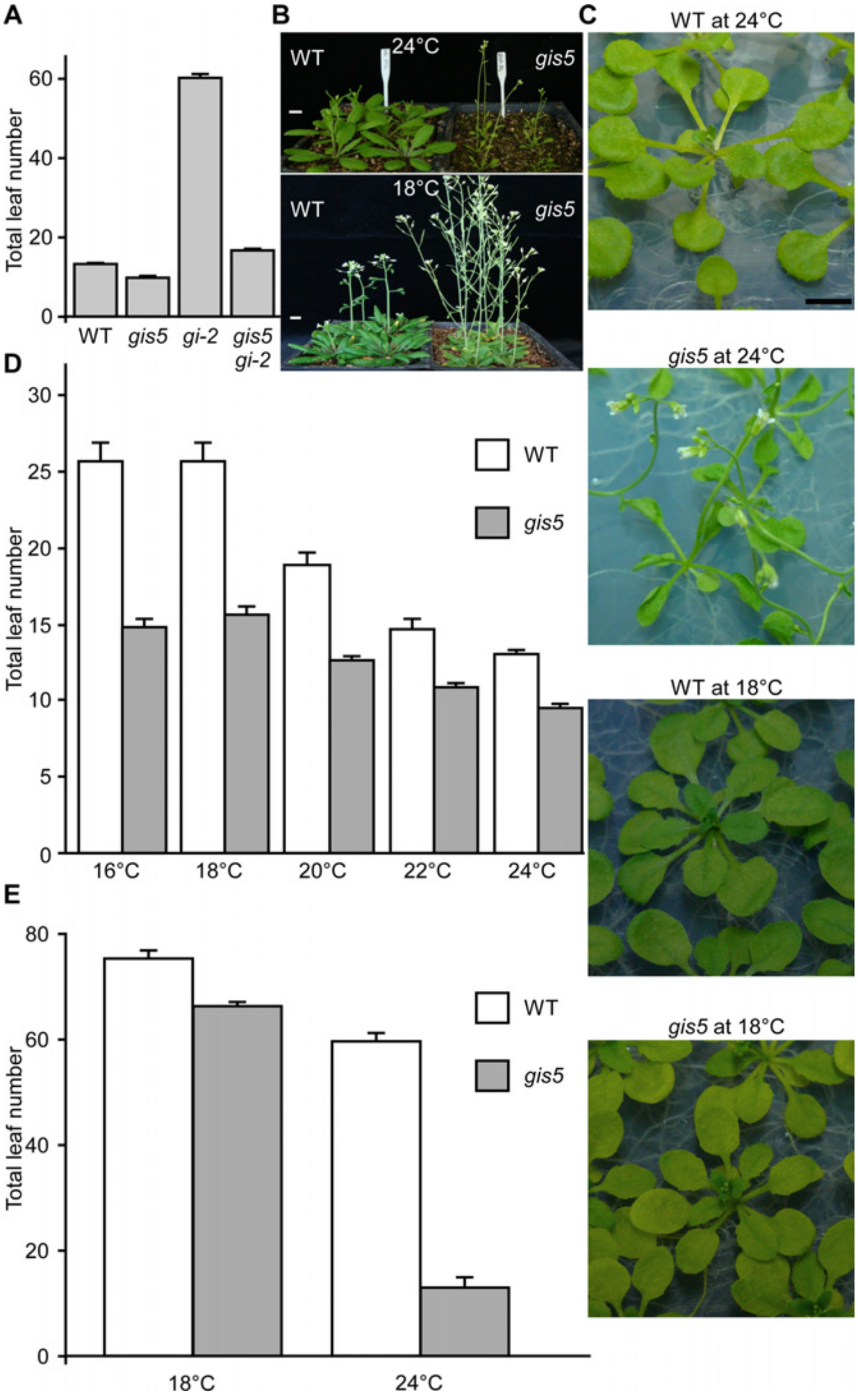

Fig 1. *gis5* phenotypes are temperature-dependent. (*A*) *gis5* mutants flower early. WT, *gis5* and *gi-2* single mutants and *gis5 gi-2* double mutant lines were grown under LD at 23°C. The total leaf number (cauline and rosette leaves) was recorded at the time of flowering. Bars represent the mean ± SEM of at least 12 plants for each genotype. (*B* and *C*) *gis5* mutants display a temperature-dependent curly leaf phenotype. WT and *gis5* mutants were grown under LD at the indicated temperatures either on soil (*B*) or MS agar plates (*C*) and photographed at flowering. Scale bar: 1 cm. (*D* and *E*) *gis5* mutants display a temperature-dependent early flowering phenotype under SD. WT and *gis5* mutants were grown under LD (*D*) or SD (*E*) at the indicated temperatures (abscissas) and flowering time was recorded as in (*A*). Bars represent the mean ± SEM of at least 12 plants for each genotype.

doi:10.1371/journal.pgen.1004975.g001

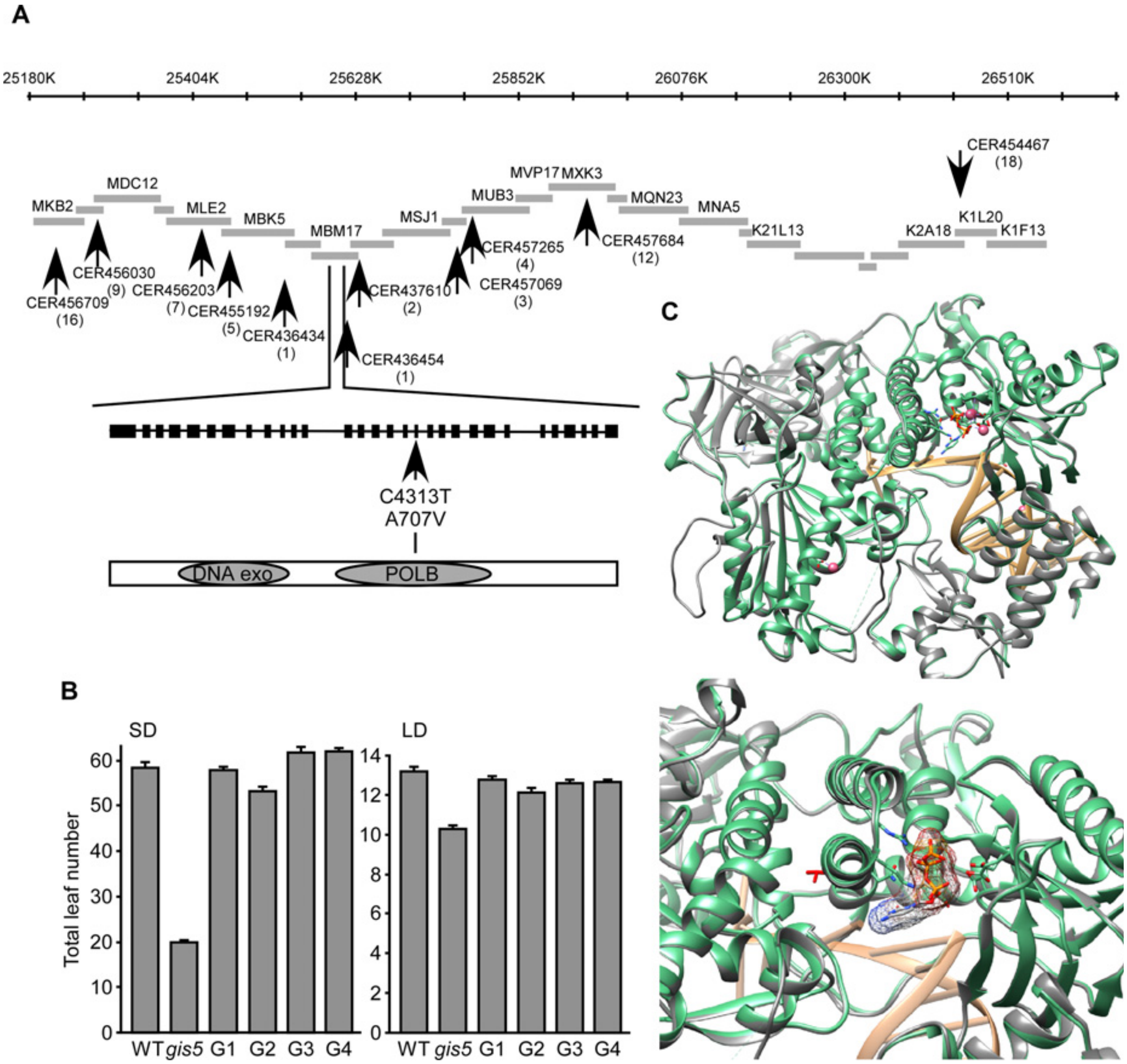

Fig 2. ***gis5*** **affects the catalytic subunit of Polδ.** (*A*) Positional cloning of *gis5*. Representation of a chromosome V interval, overlapping BACs and markers (arrows) used to screen for recombinants. The *gis5* interval is flanked by markers CER436434 and CER436454. A C>T transition was detected in the 18th exon of the At5g63960 *locus* (*POLD1*) leading to an Ala to Val substitution in the catalytic subunit of Polδ. (*B*) The WT *POLD1* sequence complements *gis5* flowering phenotype. Four independent *gis5* transgenic lines (G1 to G4), bearing a WT fragment of *POLD1*, were grown under SD (left panel) or LD (right panel) conditions. Bars represent the mean ± SEM of at least 12 plants for each genotype. (*C*) Superposition of the structural model of the catalytic subunit of Polδ and the yeast pol3 (pdb code: 3IAY). The program "Modeller" Version 9.13 [62] was used to construct the model using the X-ray structure of pol3 [27] (Top panel). Ribbon representation of the yeast Pol3, the Arabidopsis Polδ model and the DNA colored in green, gray and light orange respectively. Shown as sticks: ligand 2'-DEOXYCYTIDINE-5'-TRIPHOSPHATE (orange), 3IAY residues contacting the ligand (green). Shown as pink spheres are the Ca ions. Bottom panel: A detailed view of the modeled Polδ V707 (red sticks), which shows that the lateral chain points in the opposite direction of the substrate binding pocket.

doi:10.1371/journal.pgen.1004975.g002

The A707V Mutation May Affect Polδ Activity

The A707 residue is perfectly conserved in δ DNA polymerases from other eukaryotes (S1B Fig.). We modeled WT and mutant POLD1 using the yeast crystal structure of Pol3 as a template (PDB: 3IAY) [27]. Both proteins share 50% identity, which suggests that this particular model could be as accurate as one obtained from low resolution X-ray crystallography [28]. The A707V mutation is located in a α-helix from the finger domain (Fig. 2C) which interacts

with the nucleotide substrate during DNA polymerization. Interestingly, the A707 does not interact directly with the substrate and the A to V substitution does not affect the protein structure in any obvious manner (Fig. 2C, lower panel). Further, the A707 residue is inaccessible to the solvent, suggesting that it is not directly involved in protein to protein interactions (Fig. 2C; S1C Fig.). As the yeast Polδ conformation changes upon substrate binding [29], we modeled POLD1 in both substrate-free (4FVM) and bound (4FYD) conformations using X-Ray models from POLA1 (29% identity) as references (S1C Fig.). The α-helix bearing the A707 is greatly displaced when comparing both models, suggesting that the α-helix moves during catalysis (S1C Fig.) and that the A707V substitution may affect POLD1 activity. This was further supported by the fact that Val is known to destabilize α-helices, when replacing Ala residues [30]. Hence, the *gis5* mutation might increase the finger instability at higher temperatures.

The *gis5* Allele Is Thermosensitive

We reasoned that if the observed temperature-dependent phenotypes were due to a defect in the activity of Polδ, then we should observe similar temperature dependence on other phenotypes not related to flowering. It has been previously shown that suppression of Polδ by RNAi triggers a DNA replication stress response, including an increase in Homologous Recombination (HR) [31]. Hence, we tested if *gis5* mutants displayed a DNA replication stress response and whether this response was temperature-dependent. Interestingly, the mRNA levels of *BRCA1* and *RAD51*, two genes involved in HR, increased at 24°C in the *gis5* mutant, but only relatively weak effects were observed at 18°C (Fig. 3A). To evaluate if these changes promoted HR, we used HR reporter lines that bear halved fragments of GUS reporter genes which are reconstituted after HR events [31]. We quantified HR events, seen as blue dots after X-Gluc staining, and observed that HR events were relatively few at 18°C and greatly increased in *gis5* mutants (more than 100-fold) by growing plants at 24°C (Fig. 3B; S2 Fig.).

To further test if problems in DNA replication appeared at higher temperatures we introduced a *pCYCB1–1:GUS* reporter line in *gis5* mutants. This reporter is used to reveal cells in late G2 phases [32]. The number of GUS stained cells increased in *gis5* roots, but only at 24°C, thus suggesting that, at higher temperatures, defects in DNA replication accumulated in *gis5* cells and demanded more time for HR-dependent repair during the G2/M transition (Fig. 3C).

We reasoned that if the *gis5* allele were thermosensitive, further increasing temperature above 24°C would impair development. *gis5* mutants grown at 28°C were severely affected, resembled dwarfed plants that did not set seeds and eventually died even in axenic culture (Fig. 3D).

FT Acts Downstream of the *gis5* Mutation

To evaluate which flowering pathways are affected by the *gis5* mutation, we investigated the epistatic relationships between *gis5* and those mutations affecting the photoperiod, autonomous and vernalization pathways (Fig. 4A-B).

Mutations in the photoperiod pathway *co-9* and *gi-2* did not suppress *gis5* early flowering phenotype under both LD and SD conditions, suggesting that *gis5* is acting either downstream of these genes in the photoperiod pathway or in a parallel pathway (Fig. 4A-B).

The effects of autonomous and thermosensory pathways mutations [33], *fve-3* and *fca-9*, were mostly additive to *gis5* and the double mutants showed intermediate flowering phenotypes, suggesting that *gis5* affects parallel pathways to those affected by *fve-3* and *fca-9* (Fig. 4A-B).

FLC acts downstream of the vernalization and autonomous pathways. The *gis5* mutation produced a decrease in *FLC* mRNA levels in WT and *fca-9* backgrounds which could indicate that *gis5* might control flowering through the levels of *FLC* transcripts (S3A Fig.).

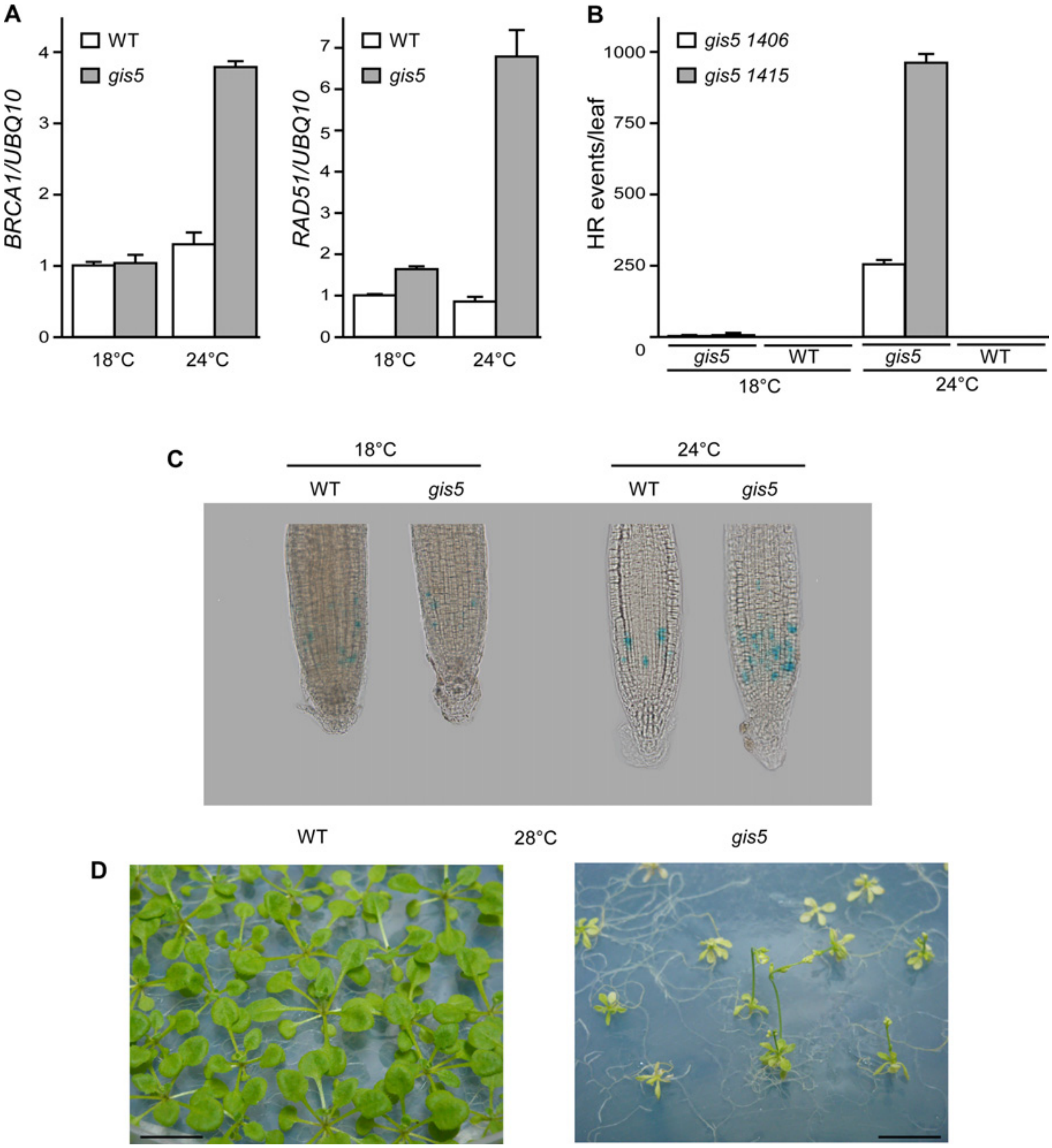

Fig 3. The *gis5* allele of the catalytic subunit of DNA polymerase δ is thermosensitive. (*A*) The *gis5* mutants display a DNA replication stress response only at higher temperatures. WT and *gis5* mutant plants were grown for 10 days under continuous light at either 18°C or 24°C. Total RNA was extracted and *BRCA1* and *RAD51* transcripts quantitated by Reverse Transcriptase-PCR (qRT-PCR) relative to *UBQ10* mRNA In each panel, WT mRNA levels at 18°C were scaled to one. Bars represent the mean ±SEM of 3 independent biological replicates, each replicate analyzed in triplicate. (*B*) Homologous Recombination (HR) increases in *gis5* mutants in a temperature-dependent manner. The *gis5* mutant was crossed with HR reporter lines bearing either direct (1415) or inverted (1406) tandem repeats of a disrupted GUS gene [31]. The repeats overlapp by 618 bp and a recombination event restores GUS activity. WT and *gis5* mutants bearing the reporter constructs were grown in LD at either 18°C or 24°C. Fully expanded 1st pair-leaves were fixed, stained with X-Gluc and photographed. Dots (HR events) were quantified as described in Materials and Methods. (*C*) *gis5* mutant cells are delayed in the G2-M transition. A *PCycB1;1*:*GUS* reporter line [32] was introduced into *gis5* mutants by crossing. Seven day old seedlings grown on LD and vertical MS agar plates at either 18 or 24°C were fixed and stained with X-gluc as described in Materials and Methods. (*D*) *gis5* mutants do not complete their life cycle at 28°C. WT (left) and *gis5* (right) mutants were grown on MS media at 28°C under LD and photographed at flowering.

doi:10.1371/journal.pgen.1004975.g003

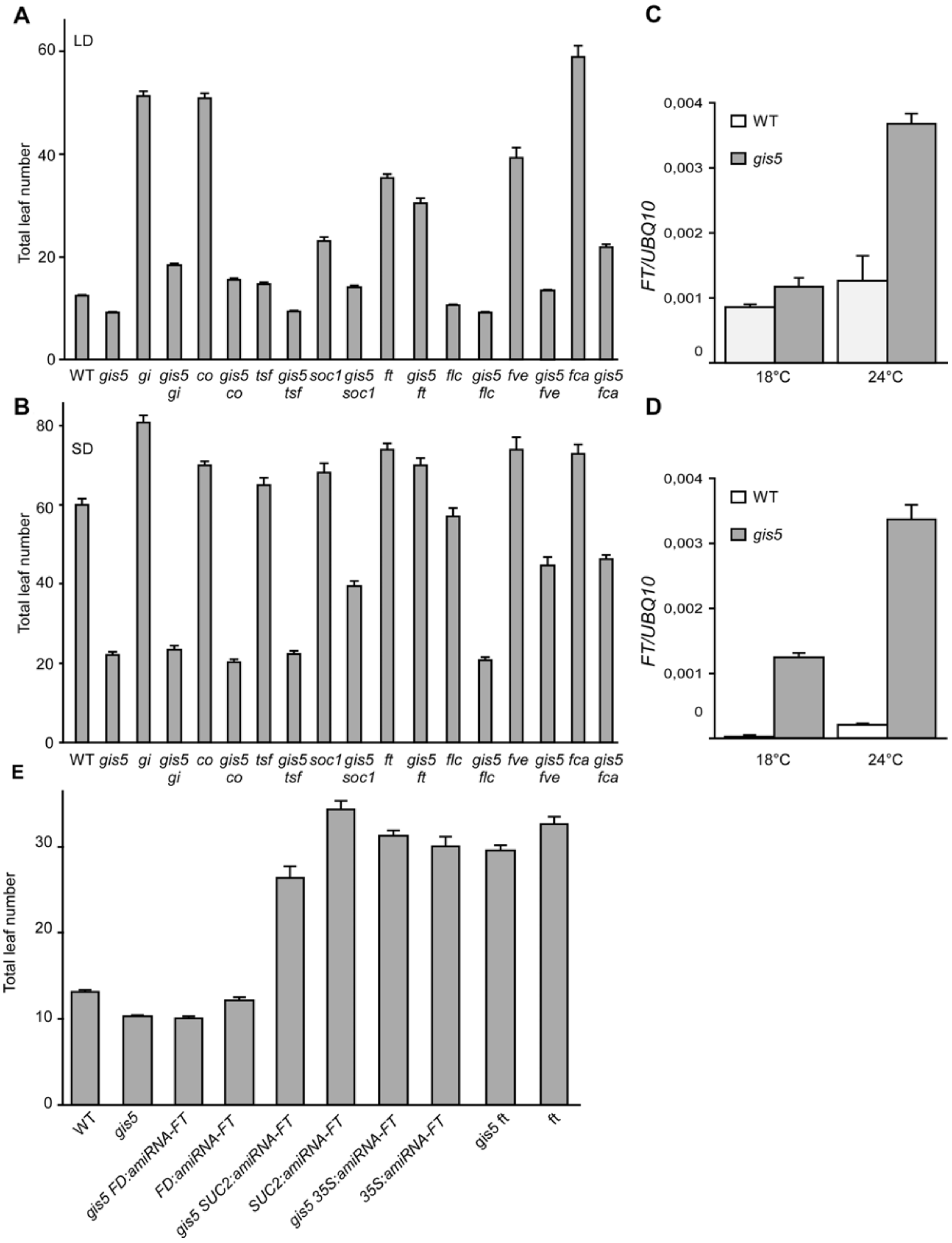

A
LD
Total leaf number
0
20
40
60
WT gis5 gi gis5 gi co gis5 co tsf gis5 tsf soc1 gis5 soc1 ft gis5 ft flc gis5 flc fve gis5 fve fca gis5 fca
B
SD
Total leaf number
0
20
40
60
80
WT gis5 gi gis5 gi co gis5 co tsf gis5 tsf soc1 gis5 soc1 ft gis5 ft flc gis5 flc fve gis5 fve fca gis5 fca
C
WT
gis5
FT/UBQ10
0
0,001
0,002
0,003
0,004
18°C
24°C
D
WT
gis5
FT/UBQ10
0
0,001
0,002
0,003
0,004
18°C
24°C
E
Total leaf number
0
10
20
30
WT
gis5
gis5 FD:amiRNA-FT
FD:amiRNA-FT
gis5 SUC2:amiRNA-FT
SUC2:amiRNA-FT
gis5 35S:amiRNA-FT
35S:amiRNA-FT
gis5 ft
ft

Fig 4. *FT* is required for *gis5* early flowering. (*A-B*) Loss of *FT* function suppresses most of *gis5* early flowering phenotype. Plants of the genotypes indicated on the abscissa were grown under LD (*A*) or SD (*B*) at 23°C and flowering recorded as in Fig. 1A. Bars represent the mean ± SEM of at least 12 plants for each genotype. (*C-D*) The increase in *FT* expression is temperature-dependent in *gis5* mutants. WT and *gis5* mutant plants were grown for 10 days under continuous light at either 18°C or 24°C (*C*), or for 21 days under SD at either temperature. Plants grown under SD were harvested at the end of the photoperiod. Total RNA was extracted and *FT* transcripts quantitated by qRT-PCR relative to *UBQ10* mRNA. Bars represent the mean ±SEM of 3 independent biological replicates, each replicate analyzed in triplicate. (*E*) Phloem specific *FT* expression is required for *gis5* early flowering. *gis5* mutants were crossed to transgenic lines bearing artificial microRNAs against *FT* expressed under specific promoters (*FD* for apical meristem specific expression, *SUC2* for phloem specific expression and *35S* for high and constitutive expression [3]. These genotypes and the corresponding controls (indicated on the abscissa) were grown under LD at 23°C and flowering recorded as in Fig. 1A. Bars represent the mean ± SEM of at least 12 plants for each genotype.

doi:10.1371/journal.pgen.1004975.g004

Notwithstanding, *gis5* mutants flowered much earlier than *flc* mutants, especially in SD (Fig. 4A-B), and the *flc* mutation did not further accelerate flowering in the *gis5* background, suggesting that *gis5* was acting mostly downstream of FLC. Hence, a role for FLC in *gis5* early flowering seemed minor in the Col background used here, but may be more important in vernalization requiring accessions. Interestingly, the *ft* mutation suppressed most of the *gis5* early flowering phenotype under both LD and SD (Fig. 4A-B), suggesting that FT acts downstream *gis5*. A mutation in the other flowering integrator gene, *suppressor of overexpression of constans 1* (*SOC1*), resulted in an intermediate effect when evaluated in the *gis5* background, which is consistent with *SOC1* being one of the downstream targets of FT [34]. A mutation in a third integrator gene, *TWIN SISTER OF FT* (*TSF*), showed marginal effects in our conditions in *gis5* and WT genetic backgrounds. Together, these results suggested that FT could be downstream of *gis5* and prompted us to measure *FT* mRNA levels. *FT* was highly expressed in the *gis5* background in both continuous light and SD (Fig. 4C-D). Further, the expression of *FT* was highly dependent on temperature in the *gis5* mutant background.

To test if ectopic or tissue specific overexpression of *FT* was related to the *gis5* phenotype, we evaluated the pattern of expression of the β-glucuronidase (GUS) reporter gene under the *FT* promoter [6]. GUS expression in *gis5 P8.1kbFT*:*GUS* plants was limited to the vascular tissue and was not detected in the apical meristem (S3B Fig.). These results, together with qRT-PCR data (Fig. 4C-D), show that *FT* is overexpressed in vascular tissue in *gis5* mutants. To test if this overexpression is functional, we used artificial microRNAs directed against *FT* mRNA, as previously reported [3]. When artificial microRNAs were expressed under the companion-cell specific promoter *SUC2*, the early flowering of *gis5* was greatly suppressed, while expression under the apical meristem specific *FD* promoter had no effect in the *gis5* mutant background (Fig. 4E). These results taken together showed that *FT* overexpression within vascular tissue was necessary for the *gis5* early flowering phenotype and were also consistent with the curly leaf phenotype observed in strong *FT* overexpressors [35].

Despite the fact that high expression of *FT* could explain both the temperature dependence and the leaf and early flowering phenotypes of *gis5* mutants, the underlying mechanism was unclear.

High Expression Levels of *SEP3* Are Temperature Dependent and Mostly FT-Independent in the *gis5* Mutant

The data presented above show that *FT* acts downstream *gis5* and is also necessary for the expression of *gis5* early flowering and curly leaf phenotypes. However, how a mutation in Polδ produced such effects was still unclear. To obtain an insight on the mechanisms, we performed a microarray analysis to study the transcriptome of *gis5* mutants to investigate if other factors could be acting upstream *FT* and be direct targets of the *gis5* allele. The genes were ordered based on the effect of *gis5* on their expression. Only a few flowering genes were found among the upregulated and downregulated genes. *SEP3* was at the top of the list, which also included *SEP1* and *SEP2* (S1 Table). Intriguingly, it has been reported that *SEP3* overexpression

accelerates flowering and leaf curling [36–38]. Further, a recent report has shown that *SEP3* can act also upstream of *FT* and that both genes mutually regulate each other in a positive manner [38]. Hence, we decided to evaluate the expression of *SEP* genes under different photoperiod and temperature conditions in *gis5* mutants. *SEP1*, *SEP2* and *SEP3* mRNAs were expressed at very high levels in the *gis5* mutants under continuous light, and this effect was highly dependent on temperature (Fig. 5), which correlates well with the phenotype of *gis5* mutants.

As FT was reported to act upstream of *SEP3* in a thermosensory pathway [39], the temperature-dependence of *gis5* phenotypes could be due to the amplification of an underlying response to temperature. We reasoned that under SD conditions and low temperatures (18°C), *SEP3* expression should be independent of both the photoperiod and the thermosensory pathways; in contrast, under elevated temperatures (24°C) the thermosensory pathway would increase *SEP3* expression in an FT-dependent manner. Hence, we also tested the expression of *SEP* genes in plants grown in SD in the *ft* mutant background. The three *SEP* genes were affected by the *ft* mutation in the *gis5* background only at 24°C and their expression dropped by about 50% in the double *gis5 ft* mutants (Fig. 5B), which is consistent with *SEP3* acting downstream FT in the thermosensory pathway [39]. However, the expression of *SEP* genes was highly elevated (30 to 100 fold) in the double *gis5 ft* mutants with respect to *ft* single mutants, revealing an FT-independent effect on *SEP* gene expression in *gis5* plants. Interestingly, despite the high expression levels of *SEP3* in *gis5* plants, FT was still required for early flowering (Fig. 4). Among the *SEP* genes, *SEP3* showed the strongest temperature response in the *gis5 ft* double mutants (4.4 fold), *SEP1* showed a partial response (1.7 fold) and *SEP2* showed no response to temperature. Taken together, these results suggested that at higher temperatures the *gis5* mutation might cause increased *FT* expression and curly leaf as well as early-flowering phenotypes by elevating *SEP3* expression.

Active Chromatin Marks in the *SEP3* Locus Increase in *gis5* Mutants in a Temperature-dependent Manner

Since the *SEP3* locus was shown to be actively repressed by H3K27me3 marks [40], we evaluated if the V707A mutation in Polδ could affect histone marks in the *SEP3* locus in a manner that is dependent on the temperature. We performed chromatin immunoprecipitation (ChIP) experiments to quantify H3K27me3, H3Ac and H3K4me3 enrichment at the *SEP3* locus in WT and *gis5* mutants grown at either 18 or 24°C. We did not find changes in H3K27me3 marks enrichment but a significant enrichment (about 5-fold) in H3K4me3 marks, which peaked in the first intron of *SEP3* and decreased towards the 3´end (Fig. 6, top right panel, S4 Fig. and S7 Fig.). Importantly, this enrichment in H3K4me3 marks on the *SEP3* locus was also dependent on temperature, showing a correlation with expression data (Fig. 5, lower left panel, Fig. 6, top panels, and S4 Fig.). H3Ac also increased in a temperature-dependent way and more likely reflects the increased transcriptional activity at the *SEP3* locus [41].

Since mutations in Polα and Polε were proposed to affect histone mark deposition at *FT* and *FLC loci* [11,16,17,19], we decided to study the deposition of histone modifications at both *loci*, in the WT and *gis5* mutants at both 18 and 24°C. Despite higher *FT* mRNA and lower *FLC* mRNA levels in *gis5* mutants, ChIP against H3Ac, H3K4me3 and H3K27me3, followed by qPCR of locus-specific fragments, did not reveal temperature-dependent changes at both the *FT* and *FLC* loci, except for a marginal increase in H3K4m3 at the *FT* locus which was not statistically significant (S5 Fig., S6 Fig. and S7 Fig). These results supported the notion that the *SEP3* locus was a primary target of a *gis5*-produced epigenetic modification.

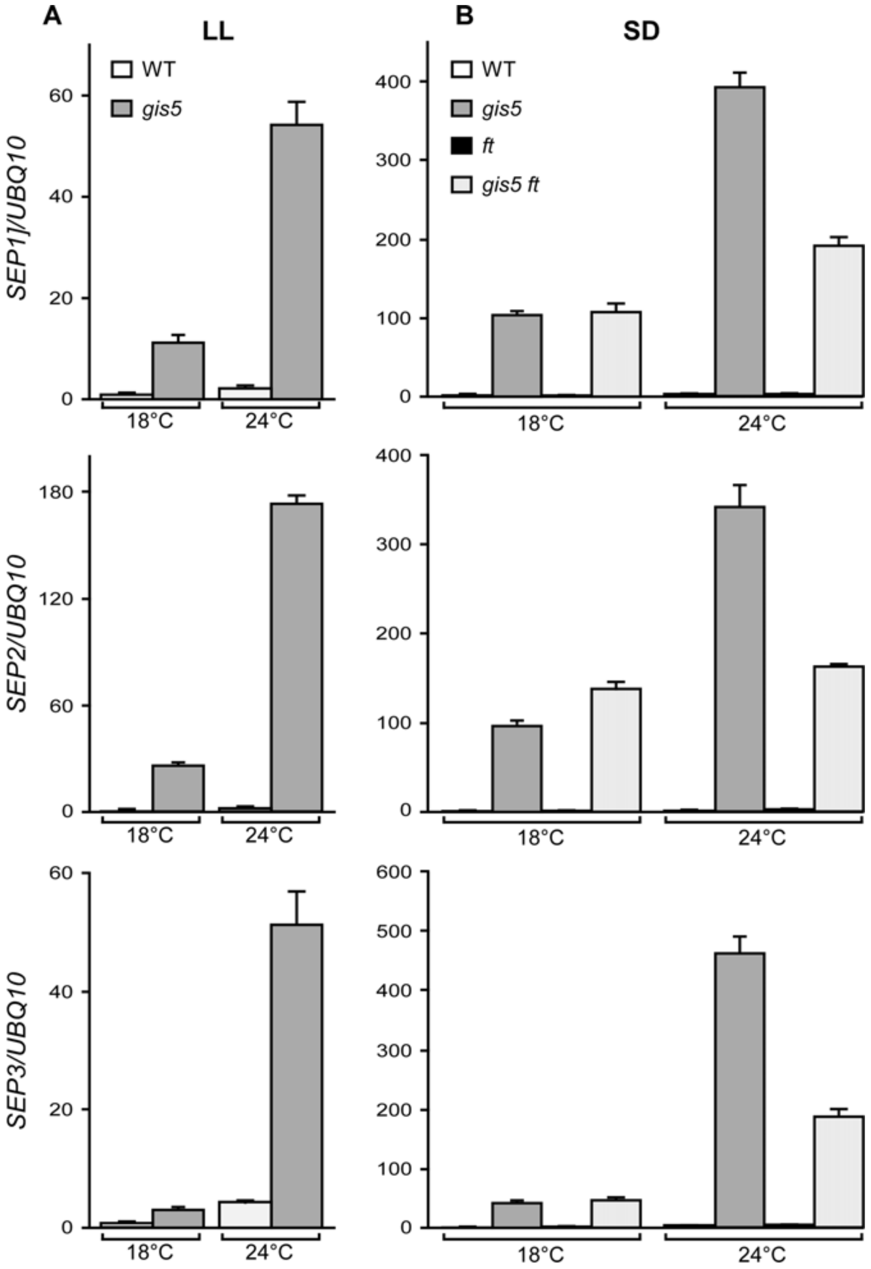

Fig 5. The expression of *SEP* genes increases in a temperature-dependent way in *gis5* mutants. (*A*-*B*) WT and *gis5* mutant plants were grown for 10 days under continuous light (*A*) at either 18°C or 24°C, or for 21 days under SD (*B*) at either temperature, together with *ft* and *gis5 ft* mutant lines. Plants grown under SD were harvested at the end of the light period. Total RNA was extracted and *SEP* transcripts quantitated by qRT-PCR relative to *UBQ10* mRNA. In each panel, WT mRNA levels at 18°C were scaled to one. Bars represent the mean ±SEM of 3 independent biological replicates, each replicate analyzed in triplicate.

doi:10.1371/journal.pgen.1004975.g005

The *gis5* Phenotype Depends on a *SEP3-FT* Feedback Loop in Phloem Tissue

To test if the elevated *SEP3* expression could be the cause of the early flowering and curly leaf phenotypes of *gis5* mutants, we suppressed *SEP3* expression in the *gis5* background using

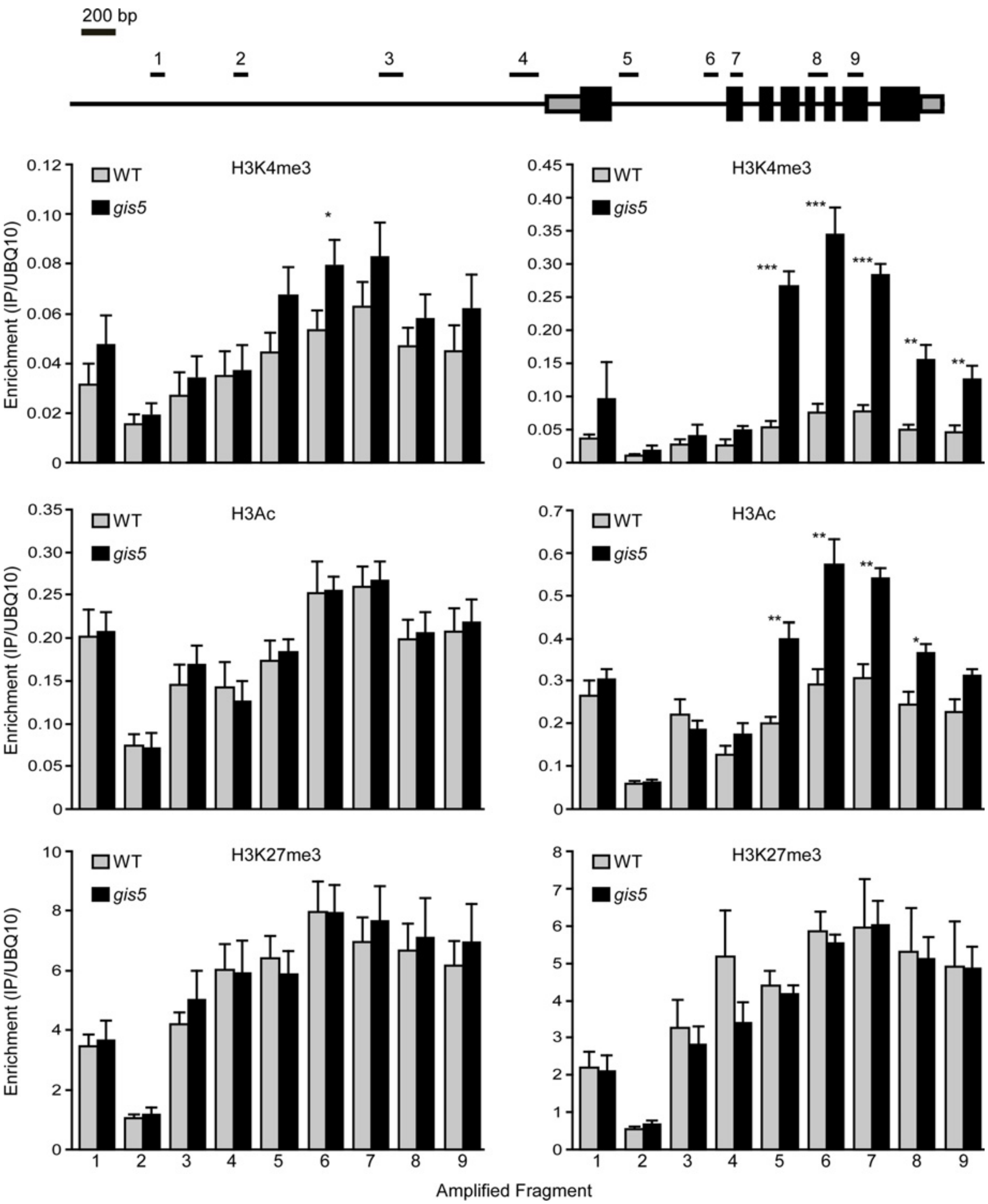

Fig 6. The *SEP3* locus is enriched in H3K4me3 and H3Ac in a temperature dependent manner. WT and *gis5* mutant plants were grown for 10 days under continuous light at either 18°C or 24°C. Enrichment in H3K4me3, H3Ac and H3K27me3 was determined by ChIP followed by qRT-PCR of the fragments depicted in the top panel. Data was relativized to a UBQ10 fragment (see S4 Fig. for data presented as a fraction of input). Bars represent the mean ± SEM of 5–6 independent biological replicates.

doi:10.1371/journal.pgen.1004975.g006

artificial microRNAs. Transgenic *gis5* lines bearing artificial microRNAs against *SEP3* showed low *SEP3* expression, decreased *FT* expression, later flowering and plain leaves (Fig. 7; S5A Fig.). On the contrary, microRNAs against *SEP1* did not show a significant effect on their own and subtle effects (if any) when coexpressed together with a microRNA against *SEP3* (Fig. 7A). Further, expression of a microRNA against *SEP3* under the phloem specific promoter *SUC2* led to a suppression of both early flowering and curly leaf phenotypes (Fig. 7) while the same microRNA under the FD promoter did not show any effect (Fig. 7C).

These pieces of evidence strongly support the notion that increased H3K4me3 marks on the *SEP3* locus and concomitant overexpression in phloem tissue are the main cause of the early flowering and leaf phenotypes of the *gis5* mutants. Conversely, the DNA replication stress response in *gis5* mutants was independent of *SEP3* expression levels, since the expression of HR marker genes *BRCA1* and *RAD51* in *gis5* background was not suppressed by suppressing *SEP3* expression (S8B Fig.). These results strongly suggest that the DNA replication stress response is activated upstream *SEP3* in *gis5* mutants and it is not a byproduct of accelerated development.

The *FLC* expression levels were lower in *gis5* mutants and interestingly, these effects were also independent of SEP3 (S8A Fig.) suggesting that FLC might have a minor SEP3-independent role in *gis5* early flowering.

The results shown above are consistent with a model where a decrease in Polδ activity in *gis5* mutants at higher temperatures leads to an increase in the expression of *SEP3*, resulting in a feedback loop with *FT* in vascular tissue to induce flowering and curly leaves phenotypes (Fig. 7D).

Despite *SEP3* overexpression can account for the *gis5* phenotypes tested here, it remains unclear whether the *gis5* mutation leads to H3K4me3 increases in other *loci*. Our microarray results showed that the expression of only 81 genes changed by at least two-fold in the *gis5* mutant with respect to the WT, suggesting that the effects of the *gis5* allele are restricted at most, to a relatively small number of *loci*. To test if other *loci* displayed changes in H3K4me3 levels, we performed ChIP experiments with a subset of genes selected from the microarray, *SEP1*, *PCC1*, and *ASN*. Both *SEP1* and *PCC1* were highy expressed (S1 Table) and also displayed a temperature-dependent increase in H3K4me3 levels (S9 Fig.), whereas *ASN* was downregulated and did not display increased H3K4me3 levels (S9 Fig.). These results show that the *gis5* allele might affect other loci diffetent from *SEP3* which may account for other phenotypes not evaluated here.

Discussion

Here we describe the isolation of a novel flowering mutant, *gis5*, which flowers early and displays curly leaves. These phenotypes are due to an A707V substitution in the catalytic subunit of the Arabidopsis Polδ encoded by the *POLD1* gene. Null *POLD1* alleles are embryo lethal (http://www.seedgenes.org/ [31]). To our knowledge, *gis5* is the first hypomorphic and viable allele to be isolated and, interestingly, it is thermosensitive. This is supported by the weak phenotypes of *gis5* mutants grown at 18°C, the strong early flowering and curly leaf phenotypes of plants grown at 24°C and the lethality of plants grown at 28°C. Further, the *gis5* mutation also produced a DNA replication stress response and an increase in HR which were also temperature-dependent. It is likely that at 18°C both Polε and Polδ advance in the replication fork in a coordinated manner. At 24°C the lower activity of the *gis5* allele would lead to larger single strand DNA stretches and eventually to DSB and increased HR, which is the mechanism to repair DSB [20]. Genetic instability was previously reported in Arabidopsis lines with low *POLD1* expression levels [31]. However, defects in epigenetic inheritance were not observed in

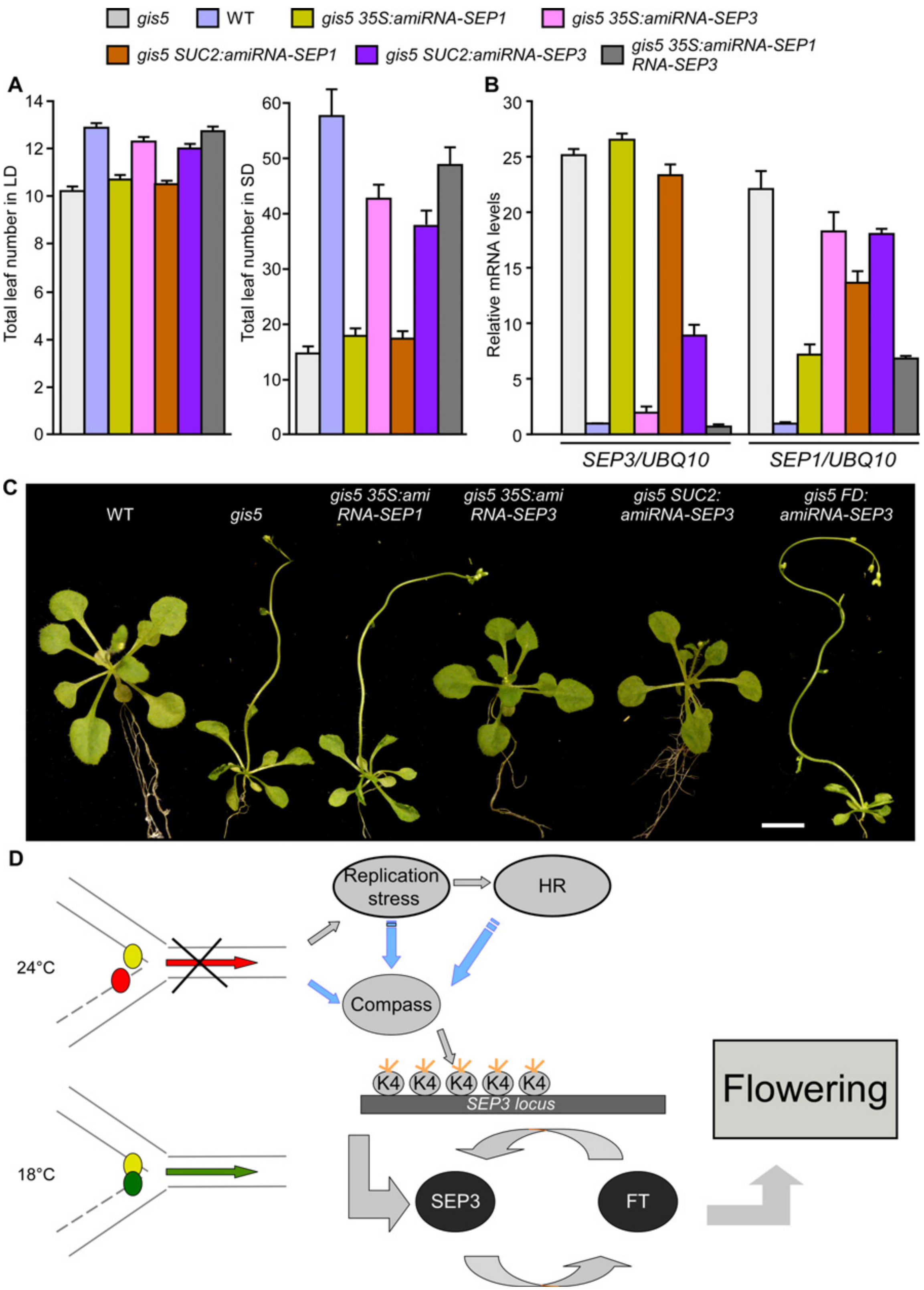

Fig 7. High levels of *SEP3* expression are required for *gis5* early flowering and leaf phenotype. (*A*) *SEP3* downregulation delays flowering in *gis5* mutants. Plants of the genotypes indicated in coloured bars were grown under either LD (left panel) or SD (right panel) at 23°C. The total leaf number (cauline and rosette leaves) was recorded at the time of flowering. Bars represent the mean ± SEM of at least 12 plants for each genotype. (*B*) *SEP3* and *SEP1* mRNA

expression levels were determined in the same genotypes shown in (*A*) as described in Fig. 5. Bars represent the mean ± SEM of 3 independent biological replicates, each replicate analyzed in triplicate. (*C*) *SEP3* downregulation suppresses *gis5* curly leaf phenotype. Plants of the indicated genotypes were grown under LD at 23°C and photographed at flowering stages. Scale bar: 1 cm. (*D*) A model explaining the temperature-dependent effect of the *gis5* allele. The replication fork is represented and works normally at 18°C (green arrow) but is stalled at 24°C (red arrow). Small colored circles represent Polε (leading strand) and Polδ (lagging strand). At higher temperatures Polδ is delayed (red circle), triggering a DNA replication stress response and increased H3K4me3 at the *SEP3* locus which participates in a positive feedback loop with *FT* to induce flowering and the curly leaf phenotype. Blue arrows indicate tentative relationships, not tested yet. The Compass complex was tentatively included because of its role in H3K4me3 establishment. Orange short lines represent trimethylation in H3K4.

doi:10.1371/journal.pgen.1004975.g007

those RNAi lines and transcriptomic analysis in those lines did not reveal changes of importance. These results, together with our data suggest that the *gis5* effects on epigenetic inheritance might result from a specific change in Polδ behavior rather than just decreased levels.

In a previous report, the incorporation of H2AZ to nucleosomes was proposed to be a mechanism of temperature perception, and its failure led to early flowering [42]. It is unlikely that a similar effect in temperature responsiveness is occurring in *gis5* mutants because the genes misexpressed in *gis5* mutants do not overlap with those misexpressed in mutants that fail to incorporate H2AZ [42]. Only one out of ten of the temperature responsive markers followed up by Kumar & Wigge (2010) showed a significant change in our microarray data of *gis5* mutants.

The early flowering and curly leaves phenotypes of *gis5* mutants are caused by high expression levels of *SEP3* which activates *FT* in phloem tissue. This is supported by our expression and genetic data and also consistent with previous reports showing that *SEP3* overexpression accelerates flowering and produces curly leaves [36–38]. *SEP3* is well known to play roles in flower development downstream of *FT* [36,37], which is also consistent with *SEP3* mRNA levels decreasing by about 50% in the *ft gis5* double mutants with respect to *gis5* single mutants. However, *SEP3* mRNA levels were still very high (about 30-fold) in *ft gis5* double mutants compared to either *ft* single mutants or WT plants indicating that *gis5* increases *SEP3* expression independently of FT. These results led us to propose that *SEP3* and *FT* form a positive feedback loop in *gis5* mutants, similar to the mutual activation of *SEP3* and *FT* reported in *clf* mutants [38], although we do not have evidence to assume that this mutual regulation is direct. The *SEP3-FT* feedback loop also explains why *gis5* early flowering is more dependent on temperature under SD compared to LD and continuous light. Under LD, *FT* mRNA levels increase in response to the photoperiod pathway contributing to the feedback loop and compensating for the decrease in *SEP3* gene activation at lower temperatures.

The strong changes in *SEP3* expression and the increases in H3K4me3 at the *SEP3* locus were both temperature-dependent and correlated well with the DNA replication stress responses and the increase in HR, suggesting that changes in *SEP3* epigenetic marks and expression were produced by the changes in the dynamics of DNA replication. Despite the fact that we cannot exclude the possibility that the effects on the *SEP3* locus were indirect (i.e. by activating a *SEP3* activator), we find this explanation rather unlikely because *i*) *SEP3* was the top upregulated gene in *gis5* mutants by our microarray data, *ii*) known *SEP3* regulators or *SEP3* coregulated genes such as *FUL* and *SPL3* were not upregulated in our microarrays and *iii*) two direct transcriptional repressors of *SEP3*, *SVP* and *AGL24* [43], were not downregulated in our microarrays. Hence, these pieces of evidence strengthen the idea that the effects of *gis5* are direct on the *SEP3* locus.

gis5 could affect *SEP3* expression by changing its pattern of histone post-translational modifications. Increased H3Ac more likely reflects the increased transcriptional activity at the *SEP3* locus [41,44]. H3K4me3 also correlates with increased transcriptional activity but it was also

shown to function as a memory of recent transcriptional activity [41,44,45]. Two scenarios are then possible, that the *gis5* mutation produces an increase in *SEP3* expression and as a consequence, an increase in H3K4me3, or that an increase in H3K4me3 causes an increase in *SEP3* transcription. We favor this second hypothesis. An increase H3K4me3 deposition at the *SEP3* locus could result from of an interaction between Polδ and the local chromatin during the maturation of Okazaki fragments. In yeast, the ligation of Okazaki fragments take place at nucleosome midpoints, implying that nucleosomes are loaded immediately after the passage of the replication fork [46]. Further, some transcription factor binding sites are also preferentially sites of Okazaki fragment ligation. These DNA bound proteins restrain excessive strand displacement by Polδ during Okazaki fragment maturation, causing Polδ to dissociate from DNA. When Polδ processivity was perturbed, the site of Okazaki fragment ligation changed consistently with Polδ dissociating before the nucleosome mid-point [46]. These data raise the possibility that the *gis5* allele of Polδ may be more sensitive to the local chromatin structure at the *SEP3* locus dissociating earlier during local Okazaki fragment maturation. If strand-displacement synthesis by Polδ is required to remove H3K4me3 incorporated during the previous Okazaki fragment synthesis, Polδ premature dissociation would eventually lead to overaccumulation of H3K4me3, which could then be involved in a positive feedback loop with transcription.

Whether the mechanisms underlying the early flowering and curly leaf phenotypes of mutants affected in Polα and Polε are similar to the mechanisms underlying *gis5* phenotypes is currently unclear. First of all, the Polδ has two chances of interacting with specific nucleosomes and DNA bound proteins, first during the extension of Okazaki fragments and then during their maturation, distinguishing Polδ from Polα and Polε. Interestingly, hypomorphic alleles of *POLA1* and *POLE1* also led to higher *SEP3* expression levels [11,16,18,19], raising the possibility that common mechanisms may produce the phenotypes of all DNA polymerase mutants. However, the role of epigenetic modifications at the *SEP3* locus were neither investigated further nor were the epistatic interactions between the *SEP3* locus and the polymerases alleles. Further, for both Polα and Polε it was proposed that their mutations affected the interaction with LIKE HETEROCHROMATIN 1 (LHP1) [11,16–19], a protein with H3K27me3 binding activity which is required to repress target genes [47,48], although some level of controversy remained on whether the proposed interactions were direct [17]. Protein modeling of the *gis5* allele suggests that the A707V substitution is unlikely to change the direct interactions with other proteins, given that this residue is not solvent accessible and very close to the nucleotide binding site, favoring the interpretation that *gis5* changes the dynamics of DNA replication rather than the recruitment of histone methylation complexes, which is supported by the expected movements of the finger domain α-helix that contains the A707V substitution. Obtaining analogous mutations to *gis5* in DNA Polα and Polε catalytic subunits will likely shed light on the possible common mechanisms that replicative polymerases may use to reestablish epigenetic marks during DNA replication.

The presence of the *SEP3-FT* feedback loop and the possible interaction of *gis5* with the *SEP3* locus accounts for the specificity of *gis5* effects. However, one remaining important question is whether the *gis5* effects are widespread all over the genome. Despite we found epigenetic changes in other loci different from *SEP3* (S9 Fig.), the relatively low number of genes whose expression is altered in *gis5* mutants (81 with a two-fold difference with WT, S1 Table), supports the notion that the effects of *gis5* are specific for a relatively low number of loci. As discussed above for the *SEP3* locus, these effects in some specific loci could result from interactions that may occur during the maturation of Okazaki fragments between Polδ and DNA bound proteins, which are loaded immediately after the passage of the replication fork [46]. Noteworthy, specific effects of mutations in the catalytic domain of Polδ are not exclusive

of plants. A high specific effect was also observed in humans; a Ser605 deletion in the Polδ catalytic site is lethal in homozygosis but heterozygous individuals showed unexpected tissue specific phenotypes: mandibular hypoplasia, deafness and progeroid features (MDP) syndrome [49]. Other mutations which affect the proofreading domains of human Polδ and Polε were associated to cancer susceptibility, which is consistent with the mutator phenotype expected for these alleles [23]. In contrast, the molecular basis for the Ser605-deletion-triggered MDP is currently unknown and there is no evidence supporting a mutator phenotype or an increase in cancer susceptibility [49]. Our work raises the possibility that an epigenetic change on a small subset of master regulators could also explain the apparent specificity of the MDP produced by deletion of Ser605 in humans, as we show here for *SEP3* in plants. In the same line of reasoning, epigenetic effects could also be part of the equation in the progression of tumors bearing defective alleles of replicative DNA polymerases, which could add to the mutator phenotypes of these defective alleles [23]. We think that the characteristics of the *gis5* allele will be invaluable in future studies on the interplay between the replication of the lagging strand, DNA replication stress, epigenetic inheritance and development in multicellular organisms.

Materials and Methods

Plant Material and Growth Conditions

All the alleles and transgenic lines used were obtained in the Columbia background: *ft-10* (GABI_290E08) [34], *tsf-3* (SALK_087522) [50], *soc1–2* (SALK_006054) [51], *co-9* (SAIL_24_HO4) [52], *gi-2* (CS3397) [53], *flc-201* (SALK_003346) [54], *fca-9* [55] and *fve-3* [56], recombination GUS reporter lines 1406 (direct repeat line) and 1415 (inverted repeat line) [57,58], *P8.1kbFT*:*GUS* [6], *PCycB1;1*:*GUS* [32], and transgenic lines expressing amiRNA-FT (artificial microRNAs) under the *35S*, *SUC2* and *FD* promoters [3]. Seeds were sterilized with chlorine in the vapour phase and, depending on the experiments, plants were grown on a 1:1:1 soil, vermiculite and perlite mix and every two weeks plants were fertilized with a 0.1% solution of Hakaphos (Compo Agricultura, http://www.compo.es), or on plates with MS salts medium (DUCHEFA). Plants were grown at 16, 18, 23, 24 and 28°C under LDs (16-h light/8-h dark), SDs (8-h light/16-h dark) or continuous light, with a light intensity of 80 μmolm^{-2}s^{-1} produced by cool white fluorescent tubes.

Map-Based Cloning and Mutant Complementation

Seeds of the late flowering *gi-2* background were mutagenized with ethyl methanesulfonate (EMS) in order to isolate early flowering, suppressors of *gi-2* mutants. The *gis5 gi-2* mutant was crossed with *gi-5* (*gi* in an Arabidopsis Landsberg erecta accession) to generate the mapping population. About 600 F2 early flowering plants were used for fine mapping by analyzing recombination events using different molecular markers (InDels and dCAPS [26,59]). The *gis5* mutation was mapped to chromosome 5 in a 120 kb interval between CER436434 and CER436454 markers. Sequencing revealed a C-to-T point mutation in the At5g63960 locus. For genetic analysis, the *gis5* mutant was backcrossed to WT four times. A fragment of 10831bp, containing the full length of At5g63960, was released from the MBM17 BAC clone by using SalI and subcloned to the pPZP212 binary vector [60]. The construct was transformed into *Agrobacterium tumefaciens* strain GV3101, which was then used to transform *gis5* mutants by floral-dipping as described [61]. The transformed seedlings were screened on MS salts plates, containing 50 mg L^{-1} kanamycin. Only homozygous, single-locus insertion lines were selected in the T3 generation and used for subsequent experiments. The primers used are described in S1 Text.

Protein Modeling

Protein structures 3IAY, that correspond to the crystal structure of the catalytic subunit of yeast Polδ in ternary complex with a template primer and an incoming nucleotide (closed conformation), 4FVM, that correspond to the catalytic subunit of yeast Polα in ternary complex with the template primer and the incoming nucleotide (closed conformation) and 4FYD, that correspond to the catalytic subunit of yeast Polα alone (open conformation), were used as templates and were obtained from the PDB website (http://www.rcsb.org/pdb/home/home.do).

Three-dimensional model of the GIS5 (wild-type and mutated) proteins were obtained by homology-modeling using Modeller V9.9 [62]. The stereochemical quality of the modelled structure was checked by assessment of the Ramachandran plot plot [63] with the rampage server (http://mordred.bioc.cam.ac.uk/~rapper/rampage.php), being 95.2% of the residues in the favoured regions. The analysis of the compatibility of the atomic model (3D) with its own amino acid sequence (1D) was performed with Verify3D [64]. Finally, the global superimposition between the template (3IAY) and the model has 881 equivalent positions with an *rmsd* of 0.43, without twists [65]. These parameters support the correctness of the model.

Genetic Analysis

Double mutants were obtained by crossing *gis5* mutants with *ft-10*, *tsf-3*, *co-9*, *gi-2*, *fve-3*, *fca-9*, *soc1–201 and flc-201* mutants. After F1 selfing, F_2 progeny was secreened by phenotyping and genotyping and verification by PCR-based methods. Primers are listed in S1 Text.

Quantitative RT-PCR

Seedlings were frozen in liquid nitrogen and total RNA was prepared using a Plant Total RNA Mini Kit (YRP50; Real Biotech Corporation, http://www.real-biotech.com), and 1 μg was used to synthesize cDNA with M-MLV reverse transcriptase (Invitrogen, http://invitrogen.com), and used to quantitate *UBQ10*, *SEP1-3*, *FT*, *FLC*, *RAD51* and *BRCA1* expression with the Mx3005P real-time PCR system (Stratagene, http://www.genomics.agilent.com) in conjunction with SyBR Green I (Invitrogen). *UBQ10* was used as a housekeeping gene to normalize gene expression [66]. Relative expression levels were determined using the comparative cycle threshold (C_t) method [67]. The primers used are described in S1 Text.

Microarray Experiments

ATH1 microarrays (Affymetrix) were used to compareWT and *gis5* transcriptomes. Total RNA was isolated, as described above, from Col and *gis5* 10-d-old seedlings grown under continuous light at 24°C. The experimental design comprised three biological replicates of each genotype. Synthesis of cDNA, cRNA labelling and hybridizations were made according to Affymetrix protocols, and probe signal intensities were processed using the Affymetrix GeneChip operating software. The resulting cell intensity (CEL) files were analyzed for data quality control using the same software package. Subsequent normalization of the raw data and estimation of signal intensities were performed using 'robust multi-chip average' (RMA) [68] with the CARMAWeb web application (https://carmaweb.genome.tugraz.at). Genes with a *P*-value lower than 0.05 and fold changes representing log2 ratio≥1 (upregulated) or ≤−1 (down regulated) were considered to be differentially expressed. The data discussed in this publication have been deposited in NCBI's Gene Expression Omnibus [69].

ChIP Assays

Three 10 cm plates of 10 day-old plants (*gis5* and Col) grown on MS agar under continuous light at 18 and 24°C were harvested and immersed in PBS supplemented with 1% formaldehyde. The seedlings were vacuum infiltrated for 20 min. Glycine was added to a final concentration of 0.1 M and incubated for 5 min. The seedlings were removed from the solution and frozen in liquid nitrogen. Approximately 2.0 g of seedlings were ground and resuspended in 25 ml NIB (50 mM HEPES [pH 7.4], 25 mM NaCl, 5 mM $MgCl_2$, 5% sucrose, 30% glycerin, 0.25% Triton X-100, 0.1% beta-mercaptoethanol, 0.1% protease inhibitor cocktail (SIGMA P9599). After centrifugation at 2500 g for 20 min at 4°C, the nuclear pellet was resuspended and washed in NWB (17 mM HEPES [pH 7.4], 7 mM $MgCl_2$, 33 mM NaCl, 13% sucrose, 13% glycerin, 0.25% Triton X-100, 0.1% beta-mercaptoethanol, 0.1% protease inhibitor cocktail). After centrifuging, the pellet was resuspended in 1mL TE buffer supplemented with 0.5% SDS and mixed on a rotator for 20 min at 4°C. The chromatin was diluted with TE buffer to a final SDS concentration of 0.25%. The DNA was sheared by sonication to approximately 500–1000 bp fragments. After centrifugation (10 min at 13,000 rpm, 4°C), approximately one tenth (4–6 μg of DNA) was mixed with RIPA dilution buffer (80 mM Tris-Hcl [pH 7.4], 230 mM NaCl, 1.7% NP40, 0.17% deoxycholate) in a 2:3 ratio and 1mM DTT, 0.5 μg/ml RNAse A, 0.2% proteinase inhibitor cocktail and 1,5 μl anti-H3K4me3 (Millipore, catalogue number: 07–473), anti-H3K27me3 (Millipore, catalogue number: 07–449) or anti-H3K9K14ac (Millipore, catalogue number: 06–599) were added. After overnight incubation with rotation at 4°C, the samples were cleared by centrifugation (14000rpm, 10 min, 4°C). A 30 μl aliquot of washed ProteinA-coupled agarose beads was added to the supernatant and the incubation continued on the rotating wheel for 1 hr at 4°C. The agarose beads were then washed with 5 times 1 mL of RIPA buffer (20 mM Tris-Hcl [pH 7.4], 140 mM NaCl, 1.0% NP40, 0.1% deoxycholate, 0.1% SDS). The immunocomplexes were eluted from the beads with two times 200 μl of glycine elution buffer (100 mM glycine, 500 mM NaCl, 0.05% Tween 20 [pH2.5]) and the combined elutes neutralized with 100 μl of 1 M Tris-HCl (pH 9.7). Crosslinks were reversed by incubation at 37°C for at least 6 hr in the presence of 60 μg/ml Proteinase K followed by at least 8 hr incubation at 65°C. The DNA was purified by two successive phenol/chloroform/isoamyl alcohol extractions and ethanol precipitation. Pellets were washed with 70% ethanol and resuspended in 100 μl of H_2O; 4 μl were used for each q-PRC. All immunoprecipitations were quantified in comparison to an appropriate dilution of the input which was obtained by processing 10% of the supernatant of each NO-antibody precipitation (only beads) in parallel to the immunoprecipitated samples during the decrosslinking and DNA purification procedure. When indicated, data was relativized to a *UBQ10* or *FUS3* fragment. Each of the immunoprecipitations was performed 5–6 independent times. The primers used are described in S1 Text.

Histochemical GUS Assays

For HR frequency determination, we counted the number of GUS positive spots, each indicating a recombination event. The recombination reporter lines 1406 (direct repeat line) and 1415 (inverted repeat line) [57,58] were crossed with *gis5*. *gis5 1406*, *gis5 1415*, *1406* and *1415* plants were grown on MS salts plates under LD conditions at 18 and 24°C. At bolting, 15 plants for each genotype and condition were dissected, and the first true leaf of 12 individual plants was used for spot number determination. A picture of each first leaf was obtained under microscope and further analyzed with the ImageJ software for spot number determination. For *FT* tissue expression studies, the *P8.1kbFTpro*:*GUS* transgenic line [6] was crossed with *gis5*, and GUS assays were performed on 10 day-old *gis5 P8.1kbFT*:*GUS* and *P8.1kbFT*:*GUS* seedlings grown on MS salts plates. For CycB1 expression analysis *gis5* was crossed with *PCycB1;1-GUS* reporter lines and GUS assays were performed as previously described [32].

amiRNA Constructs

The constructs directed against *SEP1* and *SEP3* genes were designed using WMD2 Web Micro RNA designer (http://wmd2.weigelworld.org/cgibin/mirnatools.pl; [70]). Overlapping PCR was used to replace MIR319a precursor by each microRNA and finally subcloned into pBI19 derived binary vectors for plant transformation. amiRNAs expression was driven by *35S* (ectopic expression), *SUC2* (expression in phloem companion cells) and *FD* (expression in the meristematic cells of the shoot apex). Transgenic lines were selected on medium supplemented with 50 μg mL^{-1} kanamycin. The primers used are described in S1 Text.

Accesion Numbers

Sequence data from this article can be found in GenBank/EMBL data libraries under accession numbers: At5g63960 (*POLD1*), At1g65480 (*FT*), At4g20370 (*TSF*), At2g45660 (*SOC1*), At5g15840 (*CO*), At1g22770 (*GI*), At5g10140 (*FLC*), At4g16280 (*FCA*) and At2g19520 (*FVE*). The transcriptome data can be found in GenBank (http://www.ncbi.nlm.nih.gov/geo) under Gene Expression Omnibus accession number GSE58036.

Supporting Information

S1 Fig. *gis5* affects the catalytic subunit of the Arabidopsis DNA Polymerase δ. (*A*) The WT POLD1 sequence complements *gis5* curly leaf phenotype. *gis5* mutant plants were transformed with a plasmid containing the WT POLD1 sequence (Fig. 2). T2 plants were grown on MS plates and the segregation for the T-DNA bearing the WT POLD1 sequence was analyzed. As expected, about 3/4 of the plants displayed a WT leaf phenoype. Plants not segregating for the T-DNA displayed the curly leaf phenotype typical of *gis5* mutants (arrows). (*B*) Multiple sequence alignment of the "finger B" from eukaryotic DNA polymerases. The green bar corresponds to the highly conserved distance between the K residue and the YG-pair. The K and YG-pair, in red, are fully conserved amino acids [71]. The arrow indicates the A residue mutated in *gis5*; #, essential aminoacids for DNA polymerization. Protein sequences were retrieved from SWISS-PROT (At, *Arabidopsis thaliana*; Sc, *Saccharomyces cerevisiae*; Os, *Oriza sativa*; Gm, *Glycine max*; Dd, *Dictiostelyum discoideum*; Rn, *Rattus norvergicus*; Mn, *Mus musculus*; Ma, *Mesocricetus auratus*; Bt, *Bos taurus*; Hs, *Homo sapiens*; Dm, *Drosophila melanogaster*; Ce, *Caenorabditis elegans*; Ca, *Candida albicans*; Sp, *Schizosaccharomices pombe*; Pf, *Plasmodium falciparum*. The program ClustalW2 was used for the alignment (https://www.ebi.ac.uk/Tools/msa/clustalw2/). (*C*) Substrate bound and substrate-free structural model of the catalytic subunit of DNA Polymerase d. The program "Modeller" [62] was used to construct the model using the X-ray structure of the yeast DNA Polymerase a catalytic subunit in its substrate-bound form (4FYD model, colored in light blue) and free form (4FVM model,colored in light brown). Note the displacement o the finger helix which contains Ala707 (arrows).
(TIF)

S2 Fig. The *gis5* allele of the catalytic subunit of DNA Polymerase δ is thermosensitive. The *gis5* mutants display increased HR in a temperature-dependent manner. *gis5* mutant plants were crossed into HR reporter lines 1406 and 1415. WT and *gis5* mutant plants bearing the 1406 or the 1415 reporters, as indicated, were grown in MS Agar plates under LD at either 18°C or 24°C. At bolting time, plant tissues were fixed and stained with X-Gluc. First leaves and cotyedons are shown. Dots indicate HR events which restored GUS activity [31]. Scale Bar: 1mm.
(TIF)

S3 Fig. Expression of *FT* in phloem tissue is required for *gis5* early flowering. (*A*) The gis5 mutation affects *FLC* expression. WT, *gis5*, *fve*, *fca*, *fve gis5* and *fca gis5* mutant plants were

grown for 10 days under continuous light at 24°C. Total RNA was extracted and quantitative Reverse Transcriptase-PCR (q-PCR) was performed as described in Materials and Methods to quantitate *FLC* mRNA expression relative to *UBQ10* mRNA. Bars represent the mean ±SEM of 3 independent biological replicates, each replicate analyzed in triplicate. Note that *fve* and *fve gis5* double mutants showed similar expression levels of *FLC* although they flower very differently (Fig. 3). (*B*) *FT* is expressed in vascular tissue in *gis5* mutants. *gis5* mutants were crossed into transgenic plants bearing *P8.1kbFT*:*GUS*. The 8.1 kb promoter fragment was shown to recapitulate endogenous *FT* expression patterns [6]. *gis5* (right panels) and WT (left panels) plants homozygous for the reporter construct were grown in LD at the indicated temperatures. Plants were then fixed and tissue specific GUS activity revealed with X-Guc as a substrate.
(TIF)

S4 Fig. Data from Fig. 6 represented as % of input. WT and *gis5* mutant plants were grown for 10 days under continuous light at either 18°C or 24°C. Enrichment in H3K4me3, H3Ac and H3K27me3 was determined by ChIP followed by qRT-PCR of the fragments depicted in the top panel. Bars represent the mean ± SEM of 5–6 independent biological replicates.
(TIF)

S5 Fig. The *gis5* mutant shows normal H3K4me3, H3Ac and H3K27me3 deposition on *FT* and *FLC* loci. WT and *gis5* mutant plants were grown for 10 days under continuous light at either 18°C or 24°C. Enrichment in H3K4me3, H3Ac and H3K27me3 was determined by ChIp followed by q-PCR of the fragments depicted in the top panel. Data was relativized to a *UBQ10* fragment. Bars represent the mean ±SEM of 5–6 independent biological replicates.
(TIF)

S6 Fig. Same data as in S5 Fig., but represented as % of Input. Bars represent the mean ±SEM of 5–6 independent biological replicates.
(TIF)

S7 Fig. Enrichment of H3K27me3 on loci *FLC*, *FT*, *SEP3* from Figs. 6 and S5 expressed relative to the *FUS3* fragment.
(TIF)

S8 Fig. High levels of *SEP3* expression are required for maximal *FT* expression but not for *FLC*, *BRCA1* and *RAD51* expression. (*A*) *SEP3* overexpression in phloem tissue is required for *FT* expression but not for *FLC* expression. Plants of the genotypes indicated on the abscissa were grown for 10 days under continuous light at 23°C. Total RNA was extracted and quantitative Reverse Transcriptase-PCR (q-PCR) was performed as described in Materials and Methods to quantitate *FT* (top panel) and *FLC* (lower panel). (*B*) The DNA replication stress response does not depend on *SEP3* overexpression. mRNAs for *BRCA1* (top panel) and *RAD51* (lower panel) genes were quantified as above. In each panel, WT mRNA were scaled to one. Bars represent the mean ±SEM of 3 independent biological replicates, each replicate analyzed in triplicate.
(TIF)

S9 Fig. The *gis5* effects in H3K4me3 are not unique to *SEP3*. WT and *gis5* mutant plants were grown for 10 days under continuous light at either 18°C or 24°C. Enrichment in H3K4me3 was determined by ChIP followed by qRT-PCR at the loci indicated in the abcissas. Data were expressed as a fraction of input. Bars represent the mean ± SEM of 3 independent biological replicates.
(TIF)

S1 Text. List of primers used in this study.
(PDF)

S1 Table. Expression profiling data from *gis5* and WT (Columbia background) seedlings.
(XLS)

Acknowledgments

We are grateful to the ABRC Stock Center (Ohio, USA), José A Jarillo, Ji-Hoon Ahn and Detlef Weigel for seed stocks; Barbara Hohn and David Schuermann for HR reporter lines, Peter Doerner for *CYCB1-1::GUS* lines; Markus Schmid for *FT* suppressed lines; Vanesa Gottifredi, Javier Palatnik and Maximiliano Neme for useful advice.

Author Contributions

Conceived and designed the experiments: FMI CMB FT GC PDC. Performed the experiments: FMI NAB SDD JLM. Analyzed the data: FMI CMB HL FT GC PDC. Wrote the paper: FMI PDC.

References

1. Corbesier L, Vincent C, Jang S, Fornara F, Fan Q, et al. (2007) FT protein movement contributes to long-distance signaling in floral induction of *Arabidopsis*. Science 316: 1030–1033. PMID: 17446353
2. Jaeger KE, Wigge PA (2007) FT protein acts as a long-range signal in *Arabidopsis*. Curr Biol 17: 1050–1054. PMID: 17540569
3. Mathieu J, Warthmann N, Kuttner F, Schmid M (2007) Export of FT protein from phloem companion cells is sufficient for floral induction in *Arabidopsis*. Curr Biol 17: 1055–1060. PMID: 17540570
4. Kim DH, Doyle MR, Sung S, Amasino RM (2009) Vernalization: winter and the timing of flowering in plants. Annu Rev Cell Dev Biol 25: 277–299. doi: 10.1146/annurev.cellbio.042308.113411 PMID: 19575660
5. Andres F, Coupland G (2012) The genetic basis of flowering responses to seasonal cues. Nat Rev Genet 13: 627–639. doi: 10.1038/nrg3291 PMID: 22898651
6. Adrian J, Farrona S, Reimer JJ, Albani MC, Coupland G, et al. (2010) *cis*-Regulatory elements and chromatin state coordinately control temporal and spatial expression of *FLOWERING LOCUS T* in *Arabidopsis*. Plant Cell 22: 1425–1440. doi: 10.1105/tpc.110.074682 PMID: 20472817
7. Farrona S, Thorpe FL, Engelhorn J, Adrian J, Dong X, et al. (2011) Tissue-specific expression of *FLOWERING LOCUS T* in *Arabidopsis* is maintained independently of polycomb group protein repression. Plant Cell 23: 3204–3214. doi: 10.1105/tpc.111.087809 PMID: 21917549
8. Jeong JH, Song HR, Ko JH, Jeong YM, Kwon YE, et al. (2009) Repression of *FLOWERING LOCUS T* chromatin by functionally redundant histone H3 lysine 4 demethylases in *Arabidopsis*. PloS One 4: e8033. doi: 10.1371/journal.pone.0008033 PMID: 19946624
9. Jiang D, Wang Y, He Y (2008) Repression of *FLOWERING LOCUS C* and *FLOWERING LOCUS T* by the *Arabidopsis* Polycomb repressive complex 2 components. PloS One 3: e3404. doi: 10.1371/journal.pone.0003404 PMID: 18852898
10. Wang Y, Gu X, Yuan W, Schmitz RJ, He Y (2014) Photoperiodic Control of the Floral Transition through a Distinct Polycomb Repressive Complex. Dev Cell 28: 727–736. doi: 10.1016/j.devcel.2014.01.029 PMID: 24613395
11. Barrero JM, Gonzalez-Bayon R, del Pozo JC, Ponce MR, Micol JL (2007) *INCURVATA2* encodes the catalytic subunit of DNA Polymerase alpha and interacts with genes involved in chromatin-mediated cellular memory in *Arabidopsis thaliana*. Plant Cell 19: 2822–2838. PMID: 17873092
12. Goodrich J, Puangsomlee P, Martin M, Long D, Meyerowitz EM, et al. (1997) A Polycomb-group gene regulates homeotic gene expression in *Arabidopsis*. Nature 386: 44–51. PMID: 9052779
13. Kotake T, Takada S, Nakahigashi K, Ohto M, Goto K (2003) *Arabidopsis TERMINAL FLOWER 2* gene encodes a heterochromatin protein 1 homolog and represses both *FLOWERING LOCUS T* to regulate flowering time and several floral homeotic genes. Plant Cell Physiol 44: 555–564. PMID: 12826620
14. Burgers PM (2009) Polymerase dynamics at the eukaryotic DNA replication fork. J Biol Chem 284: 4041–4045. doi: 10.1074/jbc.R800062200 PMID: 18835809

15. Li F, Martienssen R, Cande WZ (2011) Coordination of DNA replication and histone modification by the Rik1-Dos2 complex. Nature 475: 244–248. doi: 10.1038/nature10161 PMID: 21725325

16. del Olmo I, Lopez-Gonzalez L, Martin-Trillo MM, Martinez-Zapater JM, Pineiro M, et al. (2010) *EARLY IN SHORT DAYS 7 (ESD7)* encodes the catalytic subunit of DNA polymerase epsilon and is required for flowering repression through a mechanism involving epigenetic gene silencing. Plant J 61: 623–636. doi: 10.1111/j.1365-313X.2009.04093.x PMID: 19947980

17. Hyun Y, Yun H, Park K, Ohr H, Lee O, et al. (2013) The catalytic subunit of *Arabidopsis* DNA polymerase alpha ensures stable maintenance of histone modification. Development 140: 156–166. doi: 10.1242/dev.084624 PMID: 23154417

18. Liu J, Ren X, Yin H, Wang Y, Xia R, et al. (2010) Mutation in the catalytic subunit of DNA polymerase alpha influences transcriptional gene silencing and homologous recombination in *Arabidopsis*. Plant J 61: 36–45. doi: 10.1111/j.1365-313X.2009.04026.x PMID: 19769574

19. Yin H, Zhang X, Liu J, Wang Y, He J, et al. (2009) Epigenetic regulation, somatic homologous recombination, and abscisic acid signaling are influenced by DNA polymerase epsilon mutation in *Arabidopsis*. Plant Cell 21: 386–402. doi: 10.1105/tpc.108.061549 PMID: 19244142

20. Branzei D, Foiani M (2010) Maintaining genome stability at the replication fork. Nat Rev Mol Cell Biol 11: 208–219. doi: 10.1038/nrm2852 PMID: 20177396

21. Prindle MJ, Loeb LA (2012) DNA polymerase delta in DNA replication and genome maintenance. Env Mol Mutag 53: 666–682.

22. Schuermann D, Molinier J, Fritsch O, Hohn B (2005) The dual nature of homologous recombination in plants. Trends Genet 21: 172–181. PMID: 15734576

23. Palles C, Cazier JB, Howarth KM, Domingo E, Jones AM, et al. (2013) Germline mutations affecting the proofreading domains of POLE and POLD1 predispose to colorectal adenomas and carcinomas. Nat Genet 45: 136–144. doi: 10.1038/ng.2503 PMID: 23263490

24. Jenik PD, Jurkuta RE, Barton MK (2005) Interactions between the cell cycle and embryonic patterning in *Arabidopsis* uncovered by a mutation in DNA polymerase epsilon. Plant Cell 17: 3362–3377. PMID: 16278345

25. Ronceret A, Guilleminot J, Lincker F, Gadea-Vacas J, Delorme V, et al. (2005) Genetic analysis of two *Arabidopsis* DNA polymerase epsilon subunits during early embryogenesis. Plant J 44: 223–236. PMID: 16212602

26. Jander G, Norris SR, Rounsley SD, Bush DF, Levin IM, et al. (2002) Arabidopsis map-based cloning in the post-genome era. Plant Physiol 129: 440–450. PMID: 12068090

27. Swan MK, Johnson RE, Prakash L, Prakash S, Aggarwal AK (2009) Structural basis of high-fidelity DNA synthesis by yeast DNA polymerase delta. Nat Struct & Mol Biol 16: 979–986. doi: 10.1038/nsmb.2941 PMID: 25580578

28. Mullins JG (2012) Structural modelling pipelines in next generation sequencing projects. Adv Protein Chem Struct Biol 89: 117–167. doi: 10.1016/B978-0-12-394287-6.00005-7 PMID: 23046884

29. Rothwell PJ, Waksman G (2005) Structure and mechanism of DNA polymerases. Adv Protein Chem 71: 401–440. PMID: 16230118

30. Petsko GA, Ringe D (2004) Protein Structure and Function: New Science Press. PMID: 25057686

31. Schuermann D, Fritsch O, Lucht JM, Hohn B (2009) Replication stress leads to genome instabilities in *Arabidopsis* DNA polymerase delta mutants. Plant Cell 21: 2700–2714. doi: 10.1105/tpc.109.069682 PMID: 19789281

32. Colon-Carmona A, You R, Haimovitch-Gal T, Doerner P (1999) Technical advance: spatio-temporal analysis of mitotic activity with a labile cyclin-GUS fusion protein. Plant J 20: 503–508. PMID: 10607302

33. Blazquez MA, Ahn JH, Weigel D (2003) A thermosensory pathway controlling flowering time in *Arabidopsis thaliana*. Nat Genet 33: 168–171. PMID: 12548286

34. Yoo SK, Chung KS, Kim J, Lee JH, Hong SM, et al. (2005) *CONSTANS* activates *SUPPRESSOR OF OVEREXPRESSION OF CONSTANS 1* through *FLOWERING LOCUS T* to promote flowering in Arabidopsis. Plant Physiol 139: 770–778. PMID: 16183837

35. Teper-Bamnolker P, Samach A (2005) The flowering integrator *FT* regulates *SEPALLATA3* and *FRUITFULL* accumulation in *Arabidopsis* leaves. Plant Cell 17: 2661–2675. PMID: 16155177

36. Castillejo C, Romera-Branchat M, Pelaz S (2005) A new role of the *Arabidopsis SEPALLATA3* gene revealed by its constitutive expression. Plant J 43: 586–596. PMID: 16098111

37. Honma T, Goto K (2001) Complexes of MADS-box proteins are sufficient to convert leaves into floral organs. Nature 409: 525–529. PMID: 11206550

38. Lopez-Vernaza M, Yang S, Muller R, Thorpe F, de Leau E, et al. (2012) Antagonistic roles of *SEPALLATA3*, *FT* and *FLC* genes as targets of the polycomb group gene *CURLY LEAF*. PloS One 7: e30715. doi: 10.1371/journal.pone.0030715 PMID: 22363474

39. Hwan Lee J, Joon Kim J, Ahn JH (2012) Role of *SEPALLATA3 (SEP3)* as a downstream gene of *miR156-SPL3-FT* circuitry in ambient temperature-responsive flowering. Plant Signal Behav 7: 1151–1154. doi: 10.4161/psb.21366 PMID: 22899051

40. Liu C, Xi W, Shen L, Tan C, Yu H (2009) Regulation of floral patterning by flowering time genes. Dev Cell 16: 711–722. doi: 10.1016/j.devcel.2009.03.011 PMID: 19460347

41. Kim JM, To TK, Ishida J, Matsui A, Kimura H, et al. (2012) Transition of chromatin status during the process of recovery from drought stress in *Arabidopsis thaliana*. Plant Cell Physiol 53: 847–856. doi: 10.1093/pcp/pcs053 PMID: 22505693

42. Kumar SV, Wigge PA (2010) H2A.Z-containing nucleosomes mediate the thermosensory response in *Arabidopsis*. Cell 140: 136–147. doi: 10.1016/j.cell.2009.11.006 PMID: 20079334

43. Liu C, Zhou J, Bracha-Drori K, Yalovsky S, Ito T, et al. (2007) Specification of *Arabidopsis* floral meristem identity by repression of flowering time genes. Development 134: 1901–1910. PMID: 17428825

44. Ding Y, Fromm M, Avramova Z (2012) Multiple exposures to drought 'train' transcriptional responses in *Arabidopsis*. Nat Comm 3: 740.

45. Muramoto T, Muller I, Thomas G, Melvin A, Chubb JR (2010) Methylation of H3K4 Is required for inheritance of active transcriptional states. Curr Biol 20: 397–406. doi: 10.1016/j.cub.2010.01.017 PMID: 20188556

46. Smith DJ, Whitehouse I (2012) Intrinsic coupling of lagging-strand synthesis to chromatin assembly. Nature 483: 434–438. doi: 10.1038/nature10895 PMID: 22419157

47. Turck F, Roudier F, Farrona S, Martin-Magniette ML, Guillaume E, et al. (2007) *Arabidopsis* TFL2/LHP1 specifically associates with genes marked by trimethylation of histone H3 lysine 27. PLoS Genet 3: e86. PMID: 17542647

48. Zhang X, Germann S, Blus BJ, Khorasanizadeh S, Gaudin V, et al. (2007) The *Arabidopsis* LHP1 protein colocalizes with histone H3 Lys27 trimethylation. Nat Struct & Mol Biol 14: 869–871. doi: 10.1038/nsmb.2941 PMID: 25580578

49. Weedon MN, Ellard S, Prindle MJ, Caswell R, Lango Allen H, et al. (2013) An in-frame deletion at the polymerase active site of *POLD1* causes a multisystem disorder with lipodystrophy. Nat Genet 45: 947–950. doi: 10.1038/ng.2670 PMID: 23770608

50. Yamaguchi A, Kobayashi Y, Goto K, Abe M, Araki T (2005) *TWIN SISTER OF FT (TSF)* acts as a floral pathway integrator redundantly with *FT*. Plant Cell Physiol 46: 1175–1189. PMID: 15951566

51. Alonso JM, Stepanova AN, Leisse TJ, Kim CJ, Chen H, et al. (2003) Genome-wide insertional mutagenesis of *Arabidopsis thaliana*. Science 301: 653–657. PMID: 12893945

52. Balasubramanian S, Sureshkumar S, Lempe J, Weigel D (2006) Potent induction of *Arabidopsis thaliana* flowering by elevated growth temperature. PLoS Genet 2: e106. PMID: 16839183

53. Redei GP (1962) Supervital Mutants of *Arabidopsis*. Genetics 47: 443–460. PMID: 17248096

54. Strasser B, Alvarez MJ, Califano A, Cerdan PD (2009) A complementary role for ELF3 and TFL1 in the regulation of flowering time by ambient temperature. Plant J 58: 629–640. doi: 10.1111/j.1365-313X.2009.03811.x PMID: 19187043

55. Page T, Macknight R, Yang CH, Dean C (1999) Genetic interactions of the *Arabidopsis* flowering time gene *FCA*, with genes regulating floral initiation. Plant J 17: 231–239. PMID: 10097382

56. Ausin I, Alonso-Blanco C, Jarillo JA, Ruiz-Garcia L, Martinez-Zapater JM (2004) Regulation of flowering time by FVE, a retinoblastoma-associated protein. Nat Genet 36: 162–166. PMID: 14745447

57. Gherbi H, Gallego ME, Jalut N, Lucht JM, Hohn B, et al. (2001) Homologous recombination in planta is stimulated in the absence of Rad50. EMBO Rep 2: 287–291. PMID: 11306548

58. Swoboda P, Gal S, Hohn B, Puchta H (1994) Intrachromosomal homologous recombination in whole plants. EMBO J 13: 484–489. PMID: 8313893

59. Jander G (2006) Gene identification and cloning by molecular marker mapping. Methods Mol Biol 323: 115–126. PMID: 16739572

60. Hajdukiewicz P, Svab Z, Maliga P (1994) The small, versatile pPZP family of Agrobacterium binary vectors for plant transformation. Plant Mol Biol 25: 989–994. PMID: 7919218

61. Clough SJ, Bent AF (1998) Floral dip: a simplified method for Agrobacterium-mediated transformation of *Arabidopsis thaliana*. Plant J 16: 735–743. PMID: 10069079

62. Eswar N, Webb B, Marti-Renom MA, Madhusudhan MS, Eramian D, et al. (2006) Comparative protein structure modeling using Modeller. Curr Protoc Bioinformatics Chapter 5: Unit 5 6. doi: 10.1002/0471250953.bi0907s13 PMID: 18428773

63. Ramachandran GN, Ramakrishnan C, Sasisekharan V (1963) Stereochemistry of polypeptide chain configurations. J Mol Biol 7: 95–99. PMID: 13990617

64. Luthy R, Bowie JU, Eisenberg D (1992) Assessment of protein models with three-dimensional profiles. Nature 356: 83–85. PMID: 1538787

65. Ye Y, Godzik A (2003) Flexible structure alignment by chaining aligned fragment pairs allowing twists. Bioinformatics 19 Suppl 2: ii246–255. PMID: 14534198

66. Czechowski T, Stitt M, Altmann T, Udvardi MK, Scheible WR (2005) Genome-wide identification and testing of superior reference genes for transcript normalization in Arabidopsis. Plant Physiol 139: 5–17. PMID: 16166256

67. Larionov A, Krause A, Miller W (2005) A standard curve based method for relative real time PCR data processing. BMC Bioinformatics 6: 62. PMID: 15780134

68. Bolstad BM, Irizarry RA, Astrand M, Speed TP (2003) A comparison of normalization methods for high density oligonucleotide array data based on variance and bias. Bioinformatics 19: 185–193. PMID: 12538238

69. Edgar R, Domrachev M, Lash AE (2002) Gene Expression Omnibus: NCBI gene expression and hybridization array data repository. Nucleic Acids Res 30: 207–210. PMID: 11752295

70. Ossowski S, Schwab R, Weigel D (2008) Gene silencing in plants using artificial microRNAs and other small RNAs. Plant J 53: 674–690. doi: 10.1111/j.1365-313X.2007.03328.x PMID: 18269576

71. Palanivelu P (2013) DNA Polymerases: An Insight into Their Active Sites and Catalytic Mechanism. Int J Biochem Res Rev 3: 42.
